# Metabolomics, network pharmacology, and microbiome analyses uncover the mechanisms of the Chinese herbal formula for the improvement of meat quality in spent hens

**DOI:** 10.1186/s40104-025-01150-8

**Published:** 2025-02-03

**Authors:** Zhihua Li, Md. Abul Kalam Azad, Chengwen Meng, Xiangfeng Kong, Jue Gui, Wenchao Lin, Yadong Cui, Wei Lan, Qinghua He

**Affiliations:** 1 Department of Food Science and Engineering, College of Chemistry and Environmental Engineering, Shenzhen University, Shenzhen, China; 2 Hunan Provincial Key Laboratory of Animal Nutritional Physiology and Metabolic Process, National Engineering Laboratory for Pollution Control and Waste Utilization in Livestock and Poultry Production, Institute of Subtropical Agriculture, Chinese Academy of Sciences, Changsha, China; 3 School of Biology and Food Engineering, Fuyang Normal University, Fuyang, China

**Keywords:** Cecal microbiota, Chinese herbal formula, Fatty acid, Meat quality, Network pharmacology, Spent hens

## Abstract

**Background:**

Meat originating from the spent hen is an important source of poultry meat production; however, multiple factors cause the decline in the meat quality of spent hens. Chinese herbs have been widely used as medicine for a long time to prevent diseases and as nutrient supplements to improve the product quality. This experiment explored the effects of adding 1.0% Chinese herbal formula (CHF, including 0.30% *Leonurus japonicus* Houtt., 0.20% *Salvia miltiorrhiza* Bge., 0.25% *Ligustrum lucidum* Ait., and 0.25% *Taraxacum mongolicum* Hand.-Mazz.) for 120 d to the spent hens’ diet through metabolomics, network pharmacology, and microbiome strategies.

**Results:**

The results indicated that CHF supplementation improved the meat quality by reducing drip loss (*P* < 0.05), b* value (*P* = 0.058), and shear force (*P* = 0.099) and increasing cooked meat percentage (*P* = 0.054) and dry matter (*P* < 0.05) of breast muscle. The addition of CHF improved the nutritional value of breast muscle by increasing (*P* < 0.05) the content of C18:2n-6, n-6/n-3 polyunsaturated fatty acids (PUFA), total PUFA, PUFA-to-saturated fatty acids (SFA) ratio, and hypocholesterolemic-to-hypercholesterolemic ratio, and tending to increase serine content (*P* = 0.069). The targeted metabolomics analysis revealed that the biosynthesis of SFA, linoleic acid metabolism, fatty acid degradation, fatty acid elongation, and fatty acid biosynthesis pathways were enriched by CHF supplementation. Furthermore, the network pharmacology analysis indicated that CHF was closely associated with oxidative stress and lipid metabolism. The CHF supplementation increased the glutathione peroxidase level (*P* < 0.05) and upregulated gene expression related to the Nrf2 pathway (including *HO-1*, *P* < 0.05; *Nrf2*, *P* = 0.098; *CAT*, *P* = 0.060; *GPX1*, *P* = 0.063; and *SOD2*, *P* = 0.052) and lipid metabolism (including *PPARγ*, *P* < 0.05; *SREBP1*, *P* = 0.059; and *CPT1A*, *P* = 0.058). Additionally, CHF supplementation increased Firmicutes and decreased Bacteroidetes, Spirochaetes, and Synergistetes abundances (*P* < 0.05), which may contribute to better meat quality.

**Conclusions:**

Our results suggest that CHF supplementation improved the quality and nutritional value of meat, which will provide a theoretical basis for the utilization of CHF as a feed additive in spent hens’ diets.

**Graphical Abstract:**

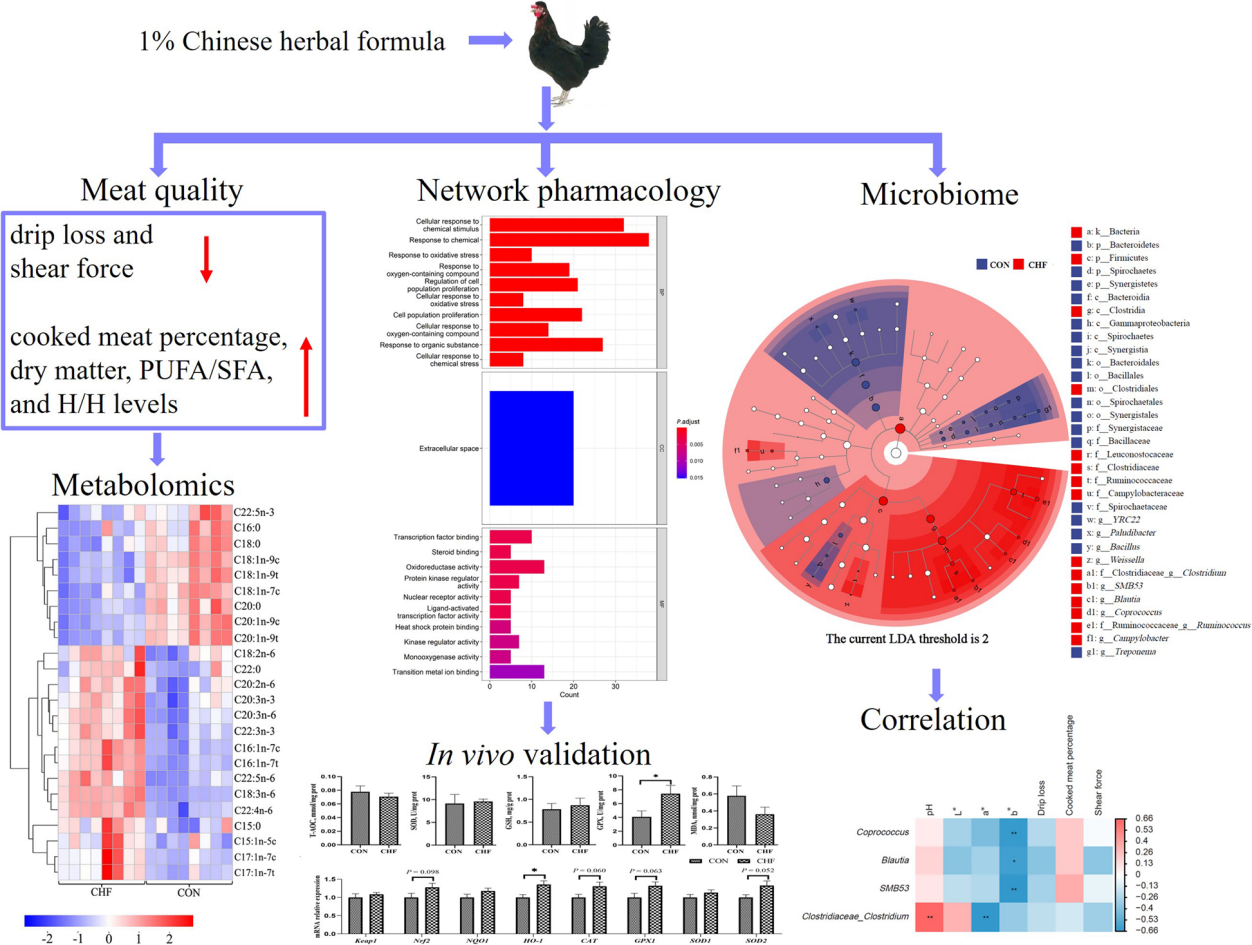

**Supplementary Information:**

The online version contains supplementary material available at 10.1186/s40104-025-01150-8.

## Background

Laying hens are generally eliminated in the layer industry due to decreased laying performance and egg quality in their final laying stage. Approximately five billion spent laying hens are discarded globally each year, of which China accounts for two billion [1]. Thus, spent hens are the primary source of chicken products besides broiler chickens. Breast muscle is one of the main economic products of spent hens; however, the meat quality, flavor, and nutritional values are relatively low, resulting in low market value of processed products, which cannot meet consumer demand [1]. Thus, formulating economically feasible and rational methods to enhance the meat quality of spent hens is a crucial aspect of the development of the layer industry. Meat quality evaluation generally includes physicochemical attributes and nutritional value [2]. Currently, studies on spent hens mainly focus on improving muscle tenderness, while little information exists on improving the nutritional value. Hence, practical strategies should be devised to improve the meat quality of spent hens.

Feed additives play crucial roles in improving the nutritional value of breast muscle in poultry nutrition and maximizing their economic potential. Chinese herbs are characterized by their advantages of affordability, widespread availability, and non-toxic side effects. The Chinese herbal formulas, as feed additives, have synergistic effects on achieving the goals of disease prevention, treatment, and nutritional supplementation due to their effective bioactive substances [3, 4]. The effective bioactive substances of Chinese herbs can enhance immunity, promote digestion and absorption, and regulate the body metabolism of animals [5]. A previous report suggested that dietary Chinese herbs or their extracts can enhance egg performance, egg quality, and plasma hormones levels of aged laying hens [6].

Multiple factors, such as systemic metabolic disorders, abnormal lipid metabolism, and antioxidant dysfunction during the late-laying stage, cause the decline in the meat quality of spent hens [7, 8]. The efficacy of individual Chinese herbs is limited. Combining multiple Chinese medicinal herbs and enhancing their synergistic effects can comprehensively regulate multiple physiological targets in various ways, resulting in favorable therapeutic outcomes. Thus, the present study selected *Leonurus japonicus* Houtt. (LJ), *Salvia miltiorrhiza* Bge. (SM), *Ligustrum lucidum* Ait. (LL), and *Taraxacum mongolicum* Hand.-Mazz. (TM) as a Chinese herb formula (CHF) based on traditional Chinese veterinary theories and specific conditions of growth and development of aged laying hens. Our previous studies found that dietary ultra-fine CHF could enhance the nutritional value of egg yolks in aged hens [9], but its potential beneficial effects on the meat quality of spent hens remain unknown.

Network pharmacology is a systematic technique to investigate potential mechanisms and functions of CHF, where the various targets and signaling pathways of CHF can be predicted [10]. The gut microbiota also regulates meat quality traits [11]. Thus, the present research focused on investigating the impacts of CHF on the quality traits and nutritional profiles of spent hens’ breast muscles. In addition, underlying mechanisms were explored using the network pharmacology, metabolomics, and microbiome strategies and validated through in vivo molecular experiments. These findings will provide a theoretical basis for improving the quality and nutritional value of spent hens’ muscles.

## Materials and methods

### Spent hens, dietary management, and sample collection

Xinyang black-feather spent hens (144 birds, 307 d old) were chosen and randomly assigned to two groups (eight replicates with nine birds per replicate). After a one-week adaptation, the birds in the control group (CON) were fed a conventional diet, while the birds in the treatment group (CHF) were fed a conventional diet supplemented with 1.0% CHF (composed of 0.30% LJ + 0.20% SM + 0.25% LL + 0.25% TM) for 120 d. The CHF was prepared as described in our previous report [9]. The conventional diet (Table S1) was formulated based on the Xinyang black-feather laying hen management guidelines (Shanghai Poultry Breeding Co., Ltd., Shanghai, China). Throughout the experiment, feeding and other management procedures were conducted following the standard protocols of the commercial poultry farm.

The laying hens were euthanized by bleeding from the jugular vein (on d 120 of the trial, 8 birds in each group), and the breast muscle samples were collected for the meat quality, nutritional value, metabolomics, and antioxidant capacity analyses. The cecal contents (2−3 cm, mid-section) were harvested for microbiome analysis.

### Meat quality analysis

Breast muscle quality indexes were detected in triplicates using the previously described methods [12]. Briefly, the pH was detected at 24 h by a pH meter (pH-STAR, Matthäus GmbH & Co. KG, Eckelsheim, Germany). A CR410 colorimeter (Konica Minolta, Inc., Tokyo, Japan) was used to assess meat color at 24 h postmortem. For drip loss analysis, breast muscle samples were trimmed into specific pieces (3 cm × 2 cm × 1 cm) and weighed before and after suspending for 24 h at 4 °C. The drip loss percentages were calculated. The breast muscle sample (15 g, refrigerated at 4 °C for 24 h) was weighed, cooked to an internal temperature of 75 °C in a water bath, dried with filter paper, and finally weighed. The cooked meat percentages were calculated. The shear force of cooked meat samples was determined with a texture analyzer (FTCTMS/PRO; Food Technology Corporation, Virginia, United States) by cutting three parallel strips vertically to the myofiber’s longitudinal axis.

### Measurement of nutritional composition

The nutritional composition of breast muscle was detected as previously described [13]. Samples were weighed, chopped, and then dried in a Scientz-100F/A vacuum-freeze dryer (Ningbo Scientz Biotechnology Co., Ltd., Ningbo, China) for 72 h to measure the dry matter (DM) content. The Kjeldahl method and Soxhlet extraction experiments were performed to detect the crude protein (CP) and intramuscular fat (IMF) levels, respectively.

### Fatty acid and amino acid profile analysis

The determination of fatty acid profiles was conducted according to the previously described method [9]. Briefly, breast muscle samples were homogenized with 50% acetonitrile-water and then centrifuged for supernatant collection. Then, the supernatant was mixed with 200 mmol/L 3-nitrophenylhydrazine and 120 mmol/L EDC (including 6% pyridine and 400 ng/µL acetic acid-d3) at a 2:1:1 (v/v/v) ratio and maintained at 40 °C for 1 h to obtain the derivatization solution. The samples were prepared by centrifugation, filtration, and dilution of the derivatization solution with 50% acetonitrile–water (including internal standard) for liquid chromatography-mass spectrometry (LC–MS) analysis with the Waters Acquity Ultra-high performance LC (Waters Corporation, Milford, MA, USA) and AB SCIEX 5,500 QTRAP-MS (AB Sciex Pte., Ltd., Framingham, MA, USA) as previously described methods [9].

For amino acid composition analysis, breast muscle samples (0.1 g) were subjected to hydrolysis with 6 mol/L hydrochloric acid (8 mL) and incubated at 110 °C for 22 h. Subsequently, the samples (1 mL) were freeze-dried at −50 °C, reconstituted in 0.2 mL of 50% acetonitrile-water solution (including 100 ng/mL tryptophan d5), agitated, sonicated at 4 °C, and then centrifuged for supernatant collection. The filtered supernatants were prepared for LC–MS analysis [3].

### Targeted metabolomics data analysis

The metabolomics analysis for fatty acids and amino acids was performed following the previously described methods [14]. Briefly, principal component analysis (PCA) and orthogonal projection to latent structures-discriminant analysis (OPLS-DA) were conducted using the SIMCA software (V14.1; Sartorius Stedim Data Analytics AB, Umea, Sweden) for visual distribution and separation of data between the two groups. The pathway enrichment analysis was conducted using the Kyoto Encyclopedia of Genes and Genomes (KEGG) and MetabolAnalyst databases.

### Network pharmacology analysis

Compounds and targets of Chinese herbs (including LJ, SM, and LL) were searched in the TCMSP database, as well as TM from the BATMAN database. Targets of four herbs were merged and deleted duplicates to obtain all targets of CHF. Additionally, the disease targets related to “fatty liver hemorrhagic syndrome” (FLHS) and “oxidative stress” (OS) were retrieved from the Genecards and the Online Mendelian Inheritance in Man databases. Redundancies were eliminated after merging the database research results to acquire all targets of FLHS or OS. The common targets of the four Chinese herbs (including LJ, SM, LL, and TM) and the diseases were filtered using the Venny 2.1. The intersections were considered potential targets for the alleviation of FLHS/OS with CHF.

Furthermore, the STRING database restricted to the “*Gallus*” species was used to construct the protein–protein interaction (PPI) network of potential targets. The herb-compound-target (H-C-T) interaction network was established through the Cytoscape (version 3.8.2). Additionally, Gene Ontology (GO) functional enrichment analysis and KEGG pathway enrichment analysis were performed using the R software (4.2.1) to decipher the mechanism of the potential targets.

### The redox status analysis

The breast muscle homogenates were prepared by mixing muscle samples (0.3 g) with normal saline (1:9, w/v), vortexing, and then centrifuging at 3,500 × *g* at 4 °C. The activities of superoxide dismutase (SOD), glutathione peroxidase (GPX), and levels of total antioxidant capacity (T-AOC), glutathione (GSH), and malondialdehyde (MDA) of muscle homogenate samples were detected using the spectrophotometer (Tecan, Infinite M200 Pro, Basel, Switzerland) following the instructions of colorimetric kits (Nanjing Jiancheng Bioengineering Institute, Nanjing, China). The content of total protein was used to normalize each index.

### RNA extraction and PCR amplification analysis

Frozen breast muscle samples were homogenized and lysed with TRIzol reagent (Accurate Biology, Changsha, China) to extract total RNA, which was then assayed for concentration and purity by a Nanophotometer N60 (Implen, GmbH, Germany) and agarose gel electrophoresis, respectively. Subsequently, reverse transcription was performed to generate cDNA following the Evo M-MLV RT Kit’s protocol (Accurate Biology, Changsha, China). Primers for the target genes were designed (Table S2) using the β-actin as the internal control gene. RT-PCR reactions were conducted on the Light CyclerR 480 II RT-PCR System (Roche, Basel, Switzerland) [14]. The relative expression levels of genes were calculated using the 2^−ΔΔCt^ method based on the acquired cycle threshold values [15].

### Cecal microbiome analysis

The Fast DNA SPIN extraction kit (MP Biomedicals, Santa Ana, CA, USA) was employed to extract the DNA from the cecal contents of spent hens. The extracted DNA was subsequently utilized for 16S V3−V4 amplification and sequencing on the IllluminaMiSeq platform (Illumina, San Diego, CA, USA) [16]. The standard protocols provided by Shanghai Personal Biotechnology Co., Ltd. (Shanghai, China) were applied for data analysis, including operational taxonomic unit (OTU) clustering, α- and β-diversity analysis, differential taxonomic analysis, and prediction of microbiota function.

### Statistical analysis

The significant differences in meat quality, nutritional values, and gene expression between the CON and CHF groups were analyzed by an independent sample *t*-test with the SPSS 22.0 software (SPSS, Inc., Chicago, IL, USA). Data are presented as mean with standard error of the mean (SEM) and considered as significant when *P* < 0.05 and a trend when 0.05 ≤ *P* < 0.10.

## Results

### The quality indicators and nutritional composition of breast muscle

Regarding the meat quality indicators of spent hens’ breast muscle (listed in Table 1), the drip loss was decreased (*P* < 0.05) and b* value (*P* = 0.058) and shear force (*P* = 0.099) showed decreasing trends in the CHF group compared with the CON group. Dietary CHF supplementation tended to increase (*P* = 0.054) cooked meat percentage. For nutritional analysis (Table 1), the addition of CHF in spent hens’ diet markedly increased (*P* < 0.05) the DM content. Meanwhile, the IMF content and CP level in the CHF group also increased in numerical value relative to the CON group.

**Table 1:** Meat quality and conventional nutritional components in breast muscle of spent hens

Items	CON group	CHF group	*P*-value
Meat quality at 24 h			
pH	5.86 ± 0.30	5.91 ± 0.33	0.272
L*	56.22 ± 0.58	55.59 ± 0.60	0.466
a*	9.98 ± 0.68	9.39 ± 0.44	0.480
b*	11.94 ± 0.50	10.37 ± 0.57	0.058
Drip loss, %	2.68 ± 0.22	1.55 ± 0.15^**^	0.001
Cooked meat percentage, %	66.56 ± 1.50	71.64 ± 1.89	0.054
Shear force, N	38.89 ± 1.68	35.08 ± 1.36	0.099
Conventional nutritional components, % of fresh weight			
Dry matter	30.71 ± 0.43	34.31 ± 1.20^*^	0.020
Intramuscular fat	6.12 ± 0.50	7.34 ± 0.59	0.133
Crude protein	24.45 ± 0.96	27.43 ± 1.94	0.174

Data are expressed as mean ± SEM (*n* = 8)

^*^P < 0.05, ^**^P < 0.01. CON group, Conventional diet; CHF group, Conventional diet supplemented with 1% Chinese herbal formula; L*, Lightness; a* , Redness; b* , Yellowness

### Fatty acid profiles in breast muscle

**Fig. 1:**
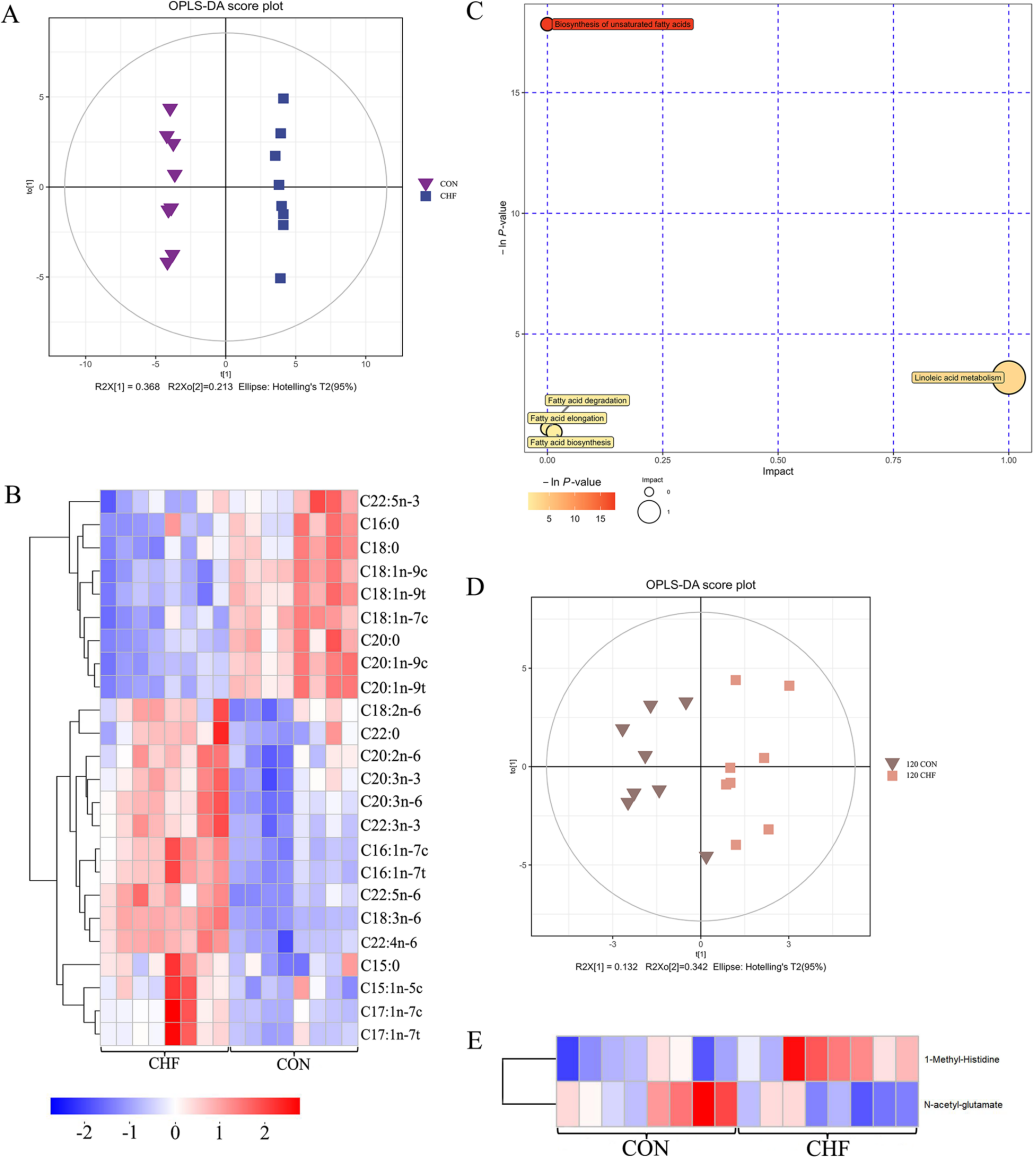
Fatty acid and amino acid profiles in breast muscle of spent hens (*n* = 8). The OPLS-DA score plots (**A**), heatmap of differential fatty acids (**B**), and enrichment of the metabolic pathways (**C**) for the CHF vs. CON groups. The OPLS-DA score plots (**D**) and heatmap of differential amino acids (**E**) for the CHF vs. CON groups. CON group, Conventional diet; CHF group, Conventional diet with the addition of 1% Chinese herbal formula; OPLS-DA, Orthogonal projections to latent structures-discriminant analysis

The PCA (Fig. S1A), OPLS-DA (Fig. 1A), and permutation test (Fig. S1B) analyses displayed the differences in fatty acid composition of breast muscle between the CHF and CON groups. The volcano plot (Fig. S1 C) and heat map (Fig. 1B) displayed that dietary CHF supplementation increased 15 and decreased 9 differential metabolites (*P* < 0.05). The results of *t*-test analysis (Table 2) showed that dietary CHF supplementation increased (*P* < 0.05) the levels of C15:0, C15:1n-5c, C16:1n-7c, C16:1n-7t, C17:1n-7c, C17:1n-7t, C18:2n-6, C18:3n-6, C20:2n-6, C20:3n-6, C20:3n-3, C22:0, C22:3n-3, C22:4n-6, and C22:5n-6 in breast muscle of spent hens compared with the CON group. The inclusion of CHF in spent hens’ diet decreased (*P* < 0.05) the levels of C16:0, C18:0, C18:1n-9c, C18:1n-9t, C18:1n-7c, C20:0, C20:1n-9c, C20:1n-9t, and C22:5n-3 relative to the CON group. Meanwhile, dietary CHF supplementation tended to increase the levels of C14:0 (*P* = 0.062) and C15:1n-5t (*P* = 0.059). The C19:1n-9t (*P* = 0.079) and C19:1n-12t (*P* = 0.069) levels displayed a decreasing trend in the CHF group relative to the CON group.

**Table 2:** Fatty acids and lipid health indices in breast muscle of spent hens, ng/mg

Item	CON	CHF	*P*-value
SFA			
C6:0	2.85 ± 0.40	2.11 ± 0.24	0.139
C7:0	0.41 ± 0.06	0.41 ± 0.06	0.982
C8:0	1.40 ± 0.33	1.52 ± 0.34	0.811
C11:0	0.11 ± 0.04	0.08 ± 0.01	0.564
C12:0	0.17 ± 0.07	0.21 ± 0.06	0.676
C14:0	3.23 ± 0.20	4.22 ± 0.44	0.062
C15:0	1.34 ± 0.07	1.61 ± 0.07^*^	0.022
C16:0	41.98 ± 0.82	38.24 ± 0.83^**^	0.006
C17:0	1.26 ± 0.04	1.32 ± 0.07	0.461
C18:0	127.23 ± 2.63	110.91 ± 2.37^***^	< 0.001
C20:0	3.35 ± 0.14	2.31 ± 0.08^***^	< 0.001
C21:0	9.44 ± 1.30	19.94 ± 7.09	0.186
C22:0	71.11 ± 0.90	74.57 ± 1.10^*^	0.029
C23:0	32.19 ± 1.64	32.31 ± 2.04	0.964
C24:0	7.17 ± 0.70	7.64 ± 1.31	0.756
MUFA			
C14:1n-5t	0.52 ± 0.07	0.54 ± 0.04	0.792
C15:1n-5c	0.24 ± 0.02	0.32 ± 0.03^*^	0.031
C15:1n-5t	0.17 ± 0.02	0.31 ± 0.06	0.059
C16:1n-7c	22.18 ± 0.47	28.54 ± 0.80^***^	< 0.001
C16:1n-7t	22.34 ± 0.45	29.19 ± 0.81^***^	< 0.001
C17:1n-7c	1.00 ± 0.03	1.30 ± 0.10^*^	0.016
C17:1n-7t	0.92 ± 0.04	1.23 ± 0.09^*^	0.015
C18:1n-9c	26.94 ± 0.40	21.80 ± 0.43^***^	< 0.001
C18:1n-7c	0.90 ± 0.02	0.65 ± 0.03^***^	< 0.001
C18:1n-9t	15.58 ± 0.27	12.99 ± 0.23^***^	< 0.001
C19:1n-9t	3.87 ± 0.11	3.62 ± 0.07	0.079
C19:1n-12t	3.31 ± 0.08	2.98 ± 0.15	0.069
C20:1n-9c	4.30 ± 0.09	3.44 ± 0.07^***^	< 0.001
C20:1n-9t	4.27 ± 0.09	3.43 ± 0.06^***^	< 0.001
C22:1n-9c	0.57 ± 0.10	0.57 ± 0.08	0.958
C22:1n-9t	0.30 ± 0.04	0.34 ± 0.10	0.718
C24:1n-9c	5.31 ± 0.07	5.35 ± 0.09	0.745
PUFA			
C18:2n-6	66.61 ± 1.08	70.62 ± 1.02^*^	0.017
C18:3n-3	1.70 ± 0.03	1.70 ± 0.17	0.994
C18:3n-6	1.32 ± 0.02	2.00 ± 0.04^***^	< 0.001
C20:2n-6	3.20 ± 0.06	3.50 ± 0.06^**^	0.003
C20:3n-6	4.49 ± 0.08	5.08 ± 0.08^***^	< 0.001
C20:3n-3	4.44 ± 0.09	4.85 ± 0.07^**^	0.002
C20:4n-6	73.45 ± 1.78	76.42 ± 1.13	0.181
C20:5n-3	0.84 ± 0.01	0.88 ± 0.02	0.146
C22:2n-6	0.32 ± 0.05	0.30 ± 0.02	0.773
C22:3n-3	13.49 ± 0.25	15.73 ± 0.33^***^	< 0.001
C22:4n-6	2.39 ± 0.07	3.17 ± 0.05^***^	< 0.001
C22:5n-6	2.93 ± 0.06	3.55 ± 0.07^***^	< 0.001
C22:5n-3	43.39 ± 0.82	40.33 ± 0.68^*^	0.012
Lipid health indices			
SFA	303.23 ± 6.21	297.39 ± 11.07	0.653
MUFA	112.72 ± 2.11	116.59 ± 2.74	0.281
PUFA	218.55 ± 4.02	228.11 ± 3.07	0.080
PUFA/SFA	0.72 ± 0.00	0.77 ± 0.03	0.088
n-3 PUFA	63.86 ± 1.12	63.48 ± 1.22	0.820
n-6 PUFA	154.69 ± 3.00	164.63 ± 2.00^*^	0.016
n-6/n-3 PUFA	2.42 ± 0.02	2.60 ± 0.03^***^	< 0.001
AI	0.17 ± 0.00	0.16 ± 0.01	0.429
TI	0.53 ± 0.00	0.46 ± 0.01^***^	< 0.001
HH	4.71 ± 0.05	5.01 ± 0.14	0.059
HPI	6.03 ± 0.09	6.30 ± 0.22	0.271
DFA	458.50 ± 8.52	455.61 ± 6.59	0.793

Data are expressed as mean ± SEM (*n* = 8)

^*^P < 0.05, ^**^P < 0.01, ^***^P < 0.001. CON group, Conventional diet; CHF group, Conventional diet with the addition of 1% Chinese herbal formula; SFA, Saturated fatty acids; MUFA, Monounsaturated fatty acids; PUFA, Polymonounsaturated fatty acids; AI, Atherogenic index; TI, Thrombopoiesis index; HH, Hypocholesterolemia/hypercholesterolemia; HPI, Health promotion index; DFA, Desirable fatty acids. The indexes were calculated as follows [9]: AI = (4 × C14:0 + C16:0)/(MUFA + PUFA), TI = (C14:0 + C16:0 + C18:0)/(0.5 × MUFA + 0.5 × n-6 PUFA + 3 × n-3 PUFA + n-3/ n-6 PUFA), HH ratio = (C18:1n-9 + C18:2n-6 + C20:4n-6 + C18:3n-3 + C20:5n-3 + C22:5n-3)/(C14:0 + C16:0), HPI = (MUFA + PUFA)/(C12:0 + 4 × C14:0 + C16:0), and DFA = C18:0 + MUFA + PUFA

Dietary CHF supplementation enriched five differential fatty acid metabolic pathways: biosynthesis of unsaturated fatty acid, linoleic acid metabolism, fatty acid degradation, fatty acid elongation, and fatty acid biosynthesis (Fig. 1C).

Additionally, regarding the lipid health indices, the inclusion of CHF in spent hens’ diet increased the n-6 polyunsaturated fatty acids (PUFA) and n-6/n-3 PUFA (*P* < 0.05), and tended to increase PUFA (*P* = 0.080), PUFA/saturated fatty acids (SFA) (*P* = 0.088), and hypocholesterolemic/hypercholesterolemic (HH) ratio (*P* = 0.059) while reduced the thrombogenic index (TI; *P *< 0.05) compared with the CON group (Table 2).

### Amino acid profiles in breast muscle

Although the PCA score plots showed no clear separation between the CHF and CON groups (Fig. S1D), the OPLS-DA and its permutation test (Fig. 1D and Fig. S1E) displayed an obvious separation between the two groups. The volcano plot (Fig. S1F) and the heat map (Fig. 1E) visualized only one increased and one decreased amino acid in the CHF group compared with the CON group. Dietary CHF significantly decreased (*P *< 0.05) the content of 1-methyl-histidine and increased (*P* < 0.05) *N*-acetyl-glutamate, while tended to increase serine (*P* = 0.069), cystine (*P* = 0.059), and pyroglutamic acid (*P* = 0.051) (Table 3). There was no pathway enrichment between the two groups.

**Table 3:** Amino acids in breast muscle of spent hens, ng/mg

Item	CON	CHF	*P*-value
EAA	1736.57 ± 111.51	1827.10 ± 153.44	0.641
Arginine	395.40 ± 30.28	429.59 ± 42.19	0.521
Histidine	227.47 ± 10.09	241.33 ± 14.83	0.453
Isoleucine	151.55 ± 11.83	159.48 ± 15.50	0.690
Leucine	151.61 ± 12.48	167.19 ± 22.66	0.557
Lysine	399.62 ± 37.81	418.78 ± 44.54	0.748
Methionine	31.26 ± 1.77	32.58 ± 2.64	0.683
Phenylalanine	182.08 ± 10.54	191.93 ± 12.31	0.553
Threonine	62.34 ± 3.84	65.12 ± 4.52	0.646
Tryptophan	83.63 ± 8.07	68.88 ± 6.91	0.187
Valine	51.61 ± 5.14	52.22 ± 4.43	0.930
NEAA	1555.30 ± 42.78	1634.39 ± 32.67	0.164
Alanine	617.03 ± 19.01	625.89 ± 16.76	0.732
Aspartic acid	50.82 ± 4.02	54.62 ± 5.04	0.565
Glutamic acid	233.43 ± 15.57	230.20 ± 15.13	0.884
Glycine	430.29 ± 30.42	484.40 ± 16.40	0.140
Proline	217.46 ± 18.83	232.21 ± 18.33	0.583
Serine	0.30 ± 0.13	1.19 ± 0.40	0.069
Tyrosine	5.97 ± 1.76	5.90 ± 1.47	0.974
Other AA			
Acetyllysine	0.68 ± 0.07	0.76 ± 0.07	0.415
Cystine	67.14 ± 2.71	73.73 ± 1.72	0.059
Dimethylglycine	3.65 ± 0.88	2.52 ± 0.50	0.282
Hydroxyproline	2.79 ± 0.30	2.89 ± 0.17	0.787
1-Methyl-Histidine	12.26 ± 0.70	9.09 ± 0.52^**^	0.003
*N*-Acetyl-glutamate	2.14 ± 0.06	2.50 ± 0.09^**^	0.006
Ornithine	75.87 ± 9.53	95.03 ± 8.32	0.152
3-Phospho-serine	746.36 ± 56.41	850.11 ± 76.79	0.295
Pyroglutamic acid	3.13 ± 0.30	4.19 ± 0.40	0.051
S-adenosyl-L-homocysteine_neg	1.19 ± 0.15	1.47 ± 0.17	0.231
S-adenosyl-L-methionine	260.12 ± 37.03	319.60 ± 36.40	0.271
Sarcosine	627.17 ± 20.00	633.59 ± 13.03	0.792
FAA	1,331.87 ± 30.11	1,396.29 ± 28.33	0.142
TAA	5,094.38 ± 212.33	5,456.96 ± 274.19	0.313

Data are expressed as mean ± SEM (*n* = 8)

^*^P < 0.05, ^**^P < 0.01. CON group, Conventional diet; CHF group, Conventional diet with the addition of 1% Chinese herbal formula; EAA, Essential amino acid, including arginine, histidine, isoleucine, leucine, lysine, methionine, phenylalanine, threonine, tryptophan, and valine. NEAA, Nonessential amino acid, including alanine, aspartic acid, glutamic acid, glycine, proline, serine, and tyrosine. FAA, Flavor amino acid, including aspartic acid, serine, glutamic acid, alanine, and glycine. TAA, Total amino acid

### Network pharmacology analysis of CHF

**Fig. 2:**
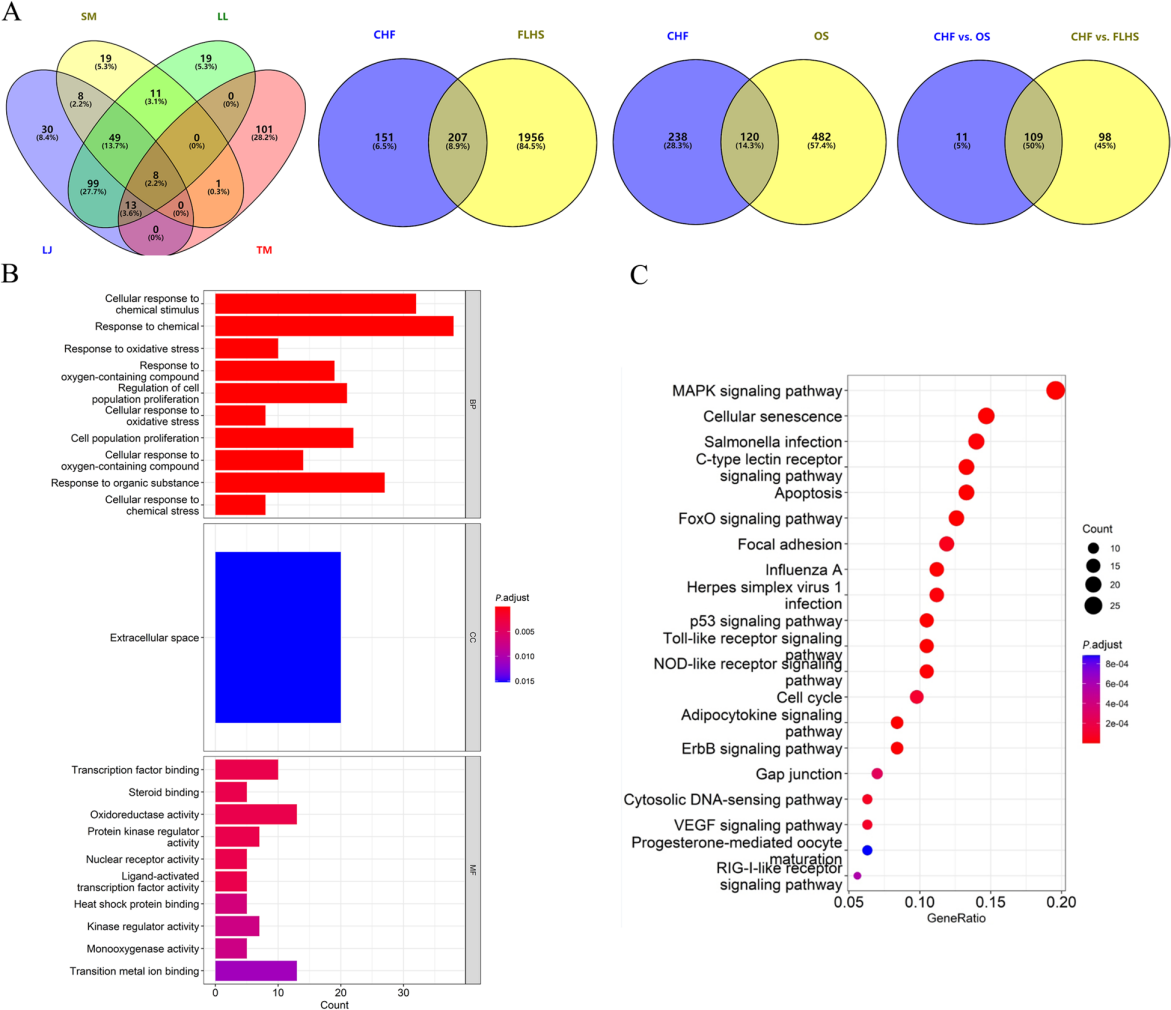
Network pharmacology analysis of effects of Chinese herbal formula (CHF) on fatty liver hemorrhagic syndrome (FLHS) and oxidative stress (OS). Venn diagrams of potential targets (**A**), enrichment analysis diagram of GO function (**B**), and KEGG pathway analysis (**C**). LJ, *Leonurus japonicus* Houtt.; SM, *Salvia miltiorrhiza* Bge.; LL, *Ligustrum lucidum* Ait.; TM, *Taraxacum mongolicum* Hand.-Mazz.; BP, Biological process; MF, Molecular function; CC, Cellular component

**Fig. 3:**
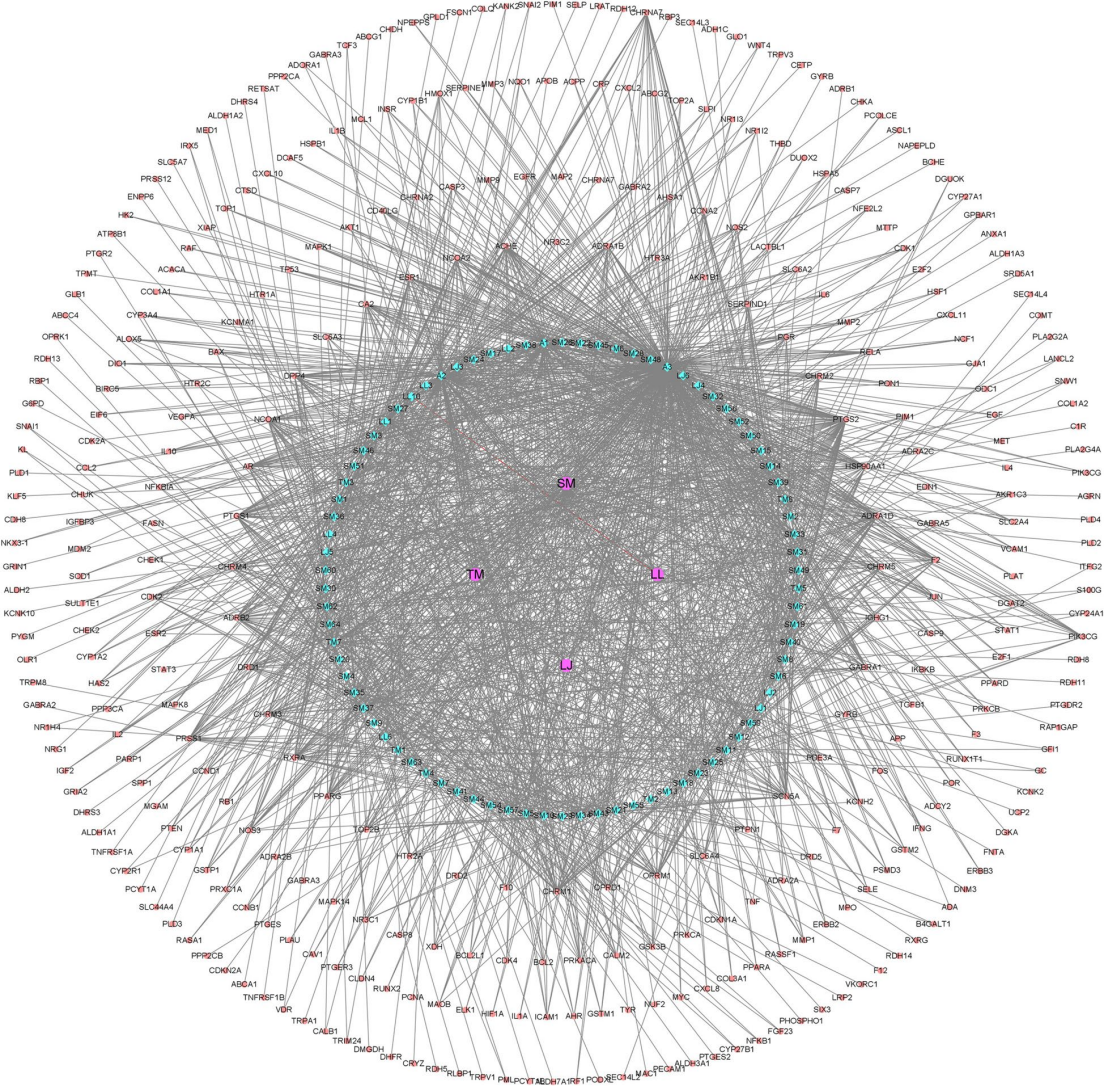
Herb-compound-target network by cytoscape. LJ, *Leonurus japonicus* Houtt.; SM, *Salvia miltiorrhiza* Bge.; LL, *Ligustrum lucidum* Ait.; TM, *Taraxacum mongolicum* Hand.-Mazz.

The results showed that there were 8, 65, and 13 pharmaceutical compounds (oral bioavailability ≥ 30% and drug-likeness ≥ 0.18) of LJ, SM, and LL from the TCMSP database, respectively. The compounds and potential targets of TM were identified by searching the BATMAN database (score cutoff ≥ 20). The bioactive compounds of four Chinese herbs included salvilenone, neocryptotanshinone, taraxerol, kaempferol, quercetin, luteolin, and others (Table S3). After removing the duplicate drug targets, a total of 358 drug targets were identified (Table S4; Fig. 2A). A total of 218 potential targets of CHF were associated with FLHS or OS (Fig. 2A). The PPI (Fig. S2) and Cytoscape (Fig. 3) analyses showed that PPARA (PPARα), PPARG (PPARγ), PPARD, NFE2L2 (Nrf2), SOD1, HMOX1 (HO-1), etc. may be the key targets for improving the meat quality of spent hens. The GO (Fig. 2B) and KEGG (Fig. 2C) results showed that steroid binding, cellular response to oxidative stress, cellular response to oxygen-containing compound, oxidoreductase activity, monooxygenase activity, and adipocytokine signaling pathways were enriched.

### The lipid metabolism and antioxidant function in breast muscle

**Fig. 4:**
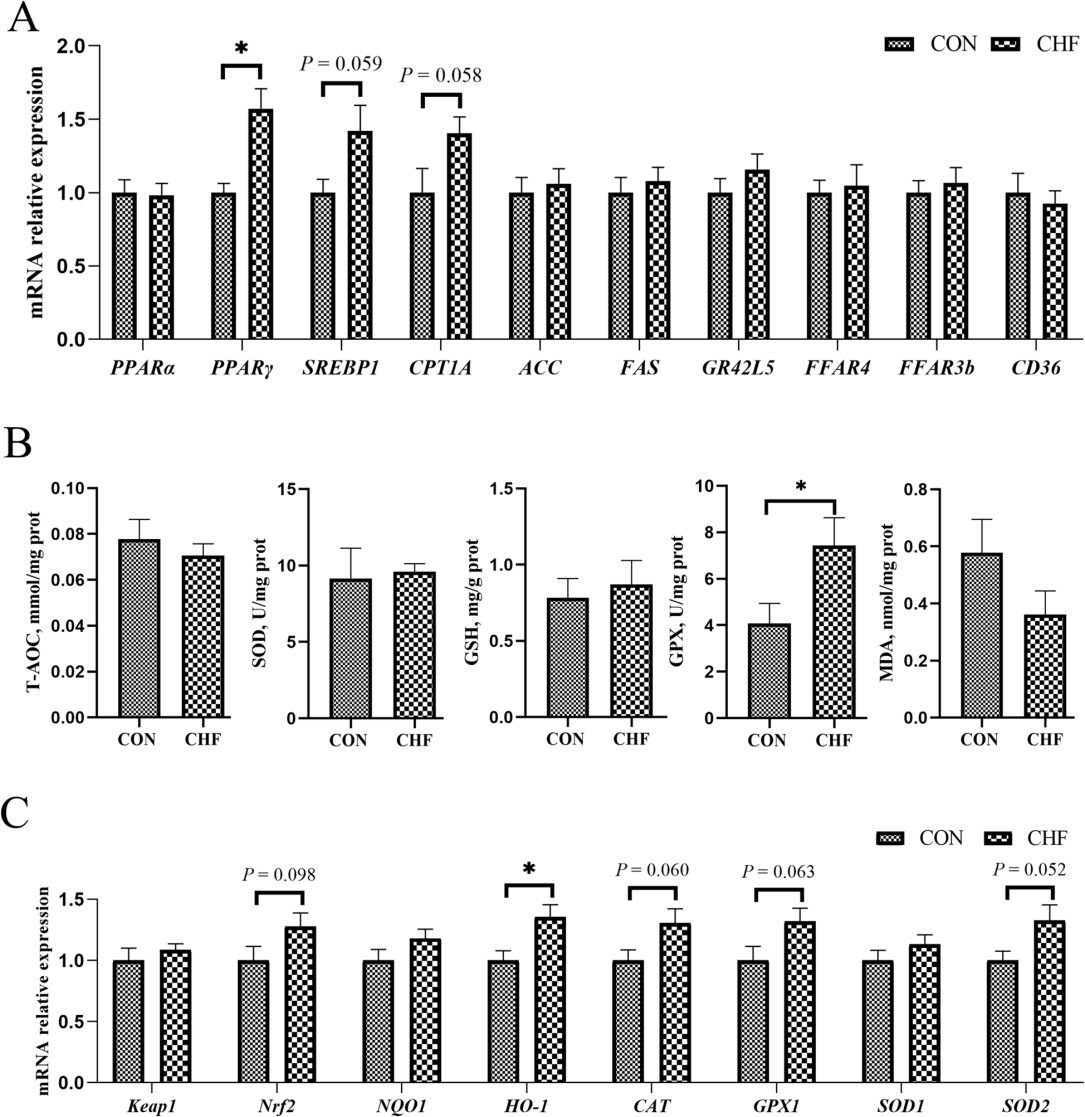
Lipid metabolism-related genes (**A**) and antioxidant function (**B** and **C**) in breast muscle of spent hens (*n* = 8). CON group, Conventional diet; CHF group, Conventional diet with the addition of 1% Chinese herbal formula. ^*^P < 0.05. *ACC*, Acetyl-CoA carboxylase; *CAT*, Catalase; *CD36*, cluster of differentiation 36; *CPT1A*, Carnitine palmitoyl transferase 1A; *FAS*, Fatty acid synthase; *FFAR3b*, Free fatty acid receptor 3b; *FFAR4*, Free fatty acid receptor 4; *GPX1*, Glutathione peroxidase 1; *GR42L5*, G-protein coupled receptor 42-like 5; *HO-1*, Heme oxygenase 1; *Keap1*, Kelch-like ECH-associated protein 1; *NQO1*, NAD(P)H quinone dehydrogenase 1; *Nrf2*, NF-E2-related factor 2; *PPARα*, Peroxisome proliferator activated receptor alpha; *PPARγ*, Peroxisome proliferator activated receptor gamma; *SOD1*, Superoxide dismutase 1; *SOD2*, Superoxide dismutase 2; *SREBP1*, Sterol regulatory element binding protein 1

The gene expression regarding lipid metabolism in breast muscle is exhibited in Fig. 4A. Dietary CHF supplementation significantly upregulated *PPARγ* (*P* < 0.05) and tended to upregulate *SREBP1* (*P* = 0.059) and *CPT1A* (*P* = 0.058) expression than the spent hens fed a conventional diet.

Regarding antioxidant function in breast muscle (Fig. 4B and 4C), spent hens supplemented with CHF had the increased GPX (*P* < 0.05) level and upregulated *HO-1* (*P* < 0.05) expression. Additionally, dietary CHF supplementation tended to upregulate *Nrf2* (*P* = 0.098), *CAT* (*P* = 0.060), *GPX1* (*P* = 0.063), and *SOD2* (*P* = 0.052) expression by comparison with the spent hens fed a conventional diet (Fig. 4C).

### Cecal microbial structure and community

**Fig. 5:**
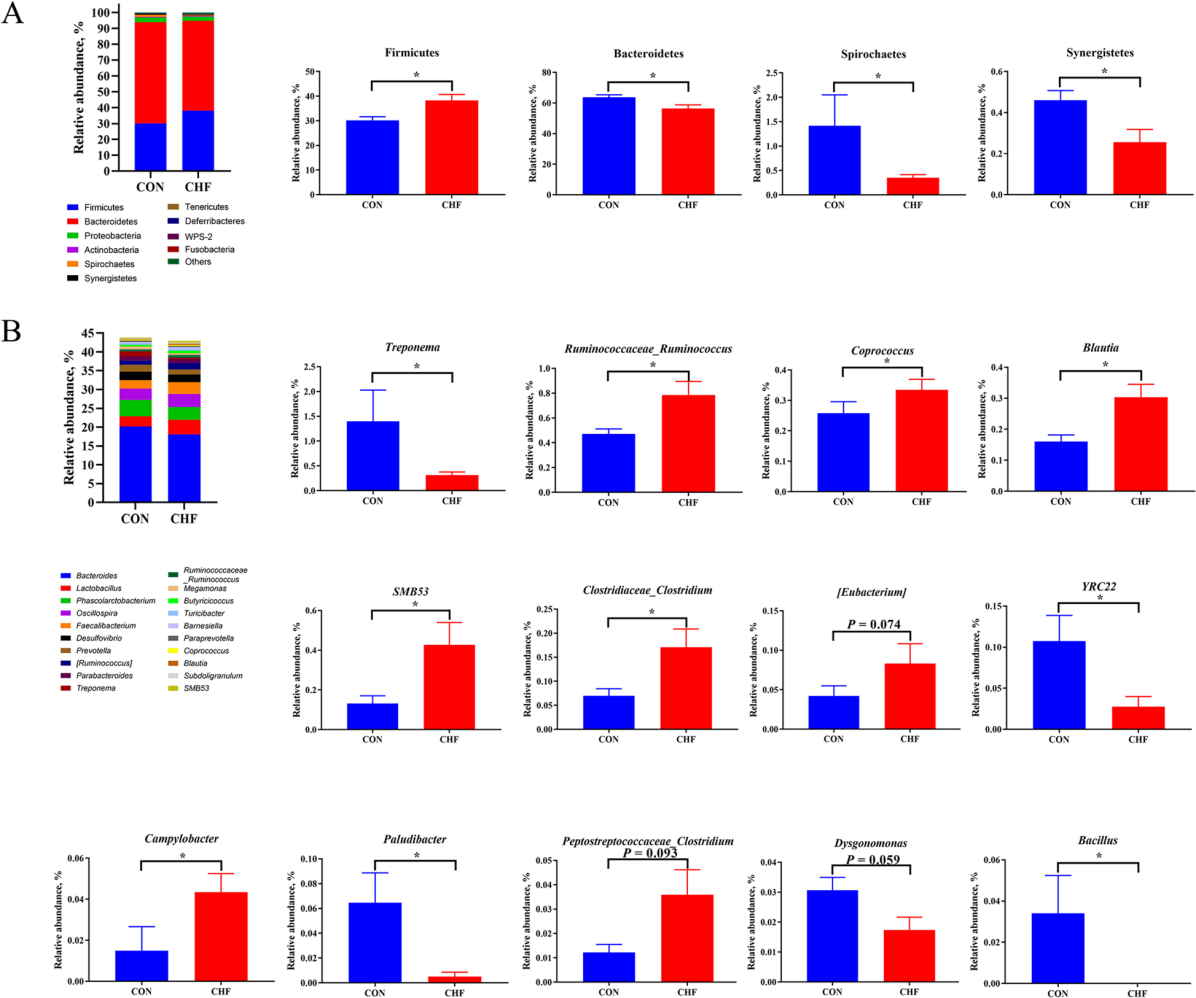
Cecal microbiota composition of spent hens (*n* = 8). Relative abundance and significant changes in cecal microbiota at the phylum (**A**) and genus (**B**) levels. ^*^P < 0.05. CON group, Conventional diet; CHF group, Conventional diet with the addition of 1% Chinese herbal formula

The α-diversity, including Chao1, Faith_pd, Observed species, and Shannon indexes, were increased (*P* < 0.05) in the CHF group than in the CON group (Fig. S3A). Although the NMDS coordination score plots (Fig. S3B) indicated that microbial communities in the cecal contents of the CHF group were not clearly detached from the CON group, the PLS-DA results (Fig. S3C) showed that microbial communities of these two groups were apart. The dominant phyla over 98% of the microbiota were Firmicutes, Bacteroidetes, Proteobacteria, Actinobacteria, Spirochaetes, and Synergistetes (Fig. 5A). Kruskal–Wallis analysis revealed that dietary CHF supplementation enhanced (*P* < 0.05) the relative abundance of Firmicutes while reduced (*P* < 0.05) the relative abundances of Bacteroidetes, Spirochaetes, and Synergistetes compared with the CON group (Fig. 5A). The Firmicutes/Bacteroidetes (F/B) ratio in the CHF group was higher (*P* < 0.05) than the CON group (Fig. S4). For the genera, the five most dominant genera, including *Bacteroides*, *Lactobacillus*, *Phascolarctobacterium*, *Oscillospira*, and *Faecalibacterium* composed over 30% of the genera in cecal contents (Fig. 5B). Dietary CHF supplementation enhanced (*P* < 0.05) the relative abundances of *Ruminococcaceae_Ruminococcus*, *Coprococcus*, *Blautia*, *SMB53*, *Clostridiaceae_Clostridium*, and *Campylobacter*, but reduced (*P* < 0.05) *Treponema*, *YRC22*, *Paludibacter*, and *Bacillus*. Meanwhile, hens supplemented with CHF tended to elevate the relative abundances of *[Eubacterium]* (*P* = 0.074) and *Peptostreptococcaceae_Clostridium* (*P* = 0.093) and reduce (*P* = 0.059) the relative abundance of *Dysgonomonas* (Fig. 5B).

### Predicted functions of different cecal microbiota

**Fig. 6:**
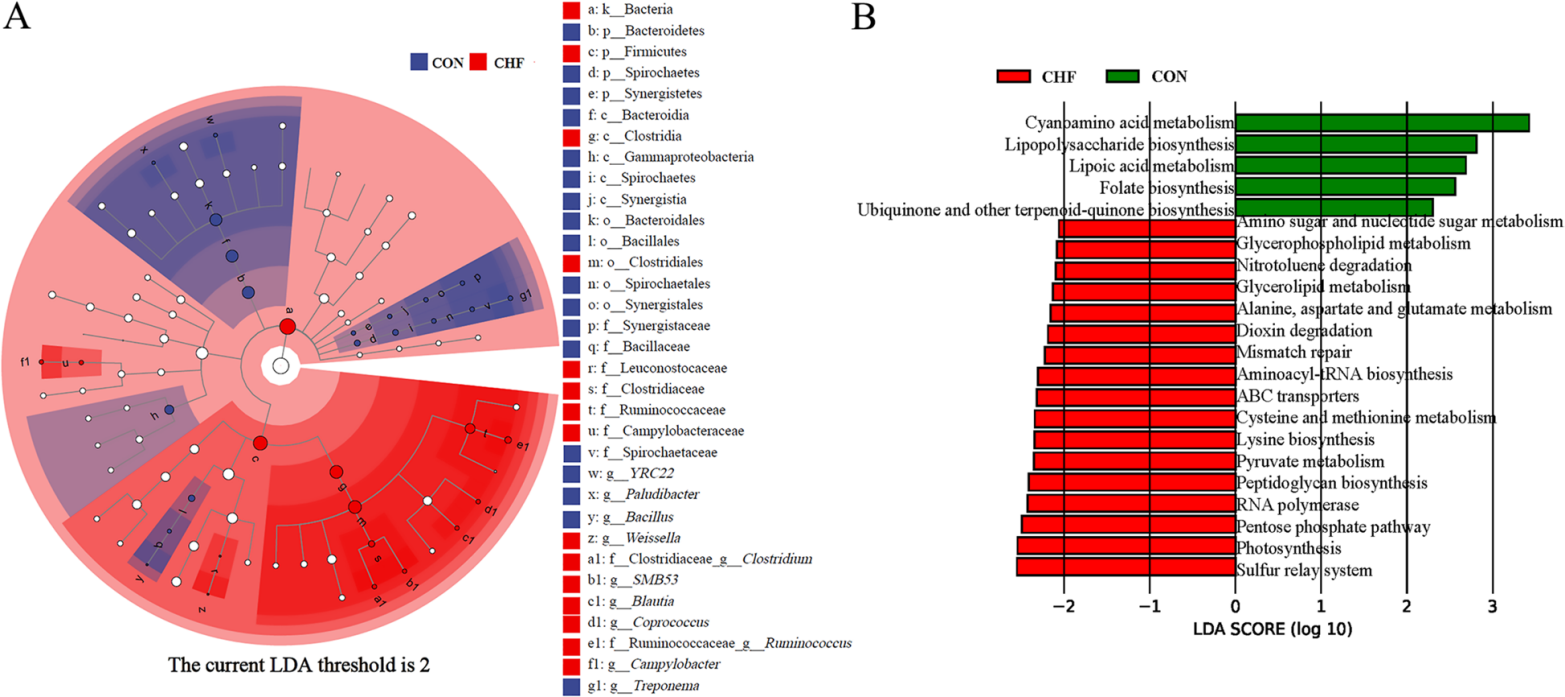
The differential cecal microbiota and pathway enrichment of spent hens (*n* = 8). Different enrichment of microbiota (**A**) and pathways at level 3 (**B**) using linear discriminant analysis (LDA) effect size (LDA score ≥ 2, *P* < 0.05). CON group, Conventional diet; CHF group, Conventional diet with the addition of 1% Chinese herbal formula

The linear discriminant analysis (LDA) effect size revealed that the CHF group enriched Firmicutes. Additionally, the CON group had comparatively higher Bacteroidetes, Spirochaetes, and Synergistetes abundances (Fig. 6A). Regarding the genus, *Weissella*, *Clostridium*, *SMB53*, *Blautia*, *Coprococcus*, *Ruminococcus*, and *Campylobacter* were enriched in the CHF group, whereas *YRC22*, *Paludibacter*, *Bacillus*, and *Treponema* were enriched in the CON group (Fig. 6A).

Phylogenetic Investigation of Communities by Reconstruction of Unobserved States (PICRUST) predictions at level 3 (Fig. 6B) showed that dietary CHF reduced (LDA > 2, *P* < 0.05) pathways related to the microbial-predicted metabolism: cyanoamino acid metabolism, lipopolysaccharide biosynthesis, lipoic acid metabolism, folate biosynthesis, ubiquinone, and other terpenoid-quinone biosynthesis. Moreover, CHF supplementation enriched the microbial-predicted metabolism pathways related to amino sugar and nucleotide sugar metabolism, glycerophospholipid metabolism, nitrotoluene degradation, glycerolipid metabolism, alanine/aspartate/glutamate metabolism, dioxin degradation, mismatch repair, aminoacyl-tRNA biosynthesis, ABC transporters, cysteine and methionine metabolism, lysine biosynthesis, pyruvate metabolism, peptidoglycan biosynthesis, RNA polymerase, pentose phosphate pathway, photosynthesis, and sulfur relay system.

### Correlations among meat quality, fatty acids, and differentiated cecal microbiota

**Fig. 7:**
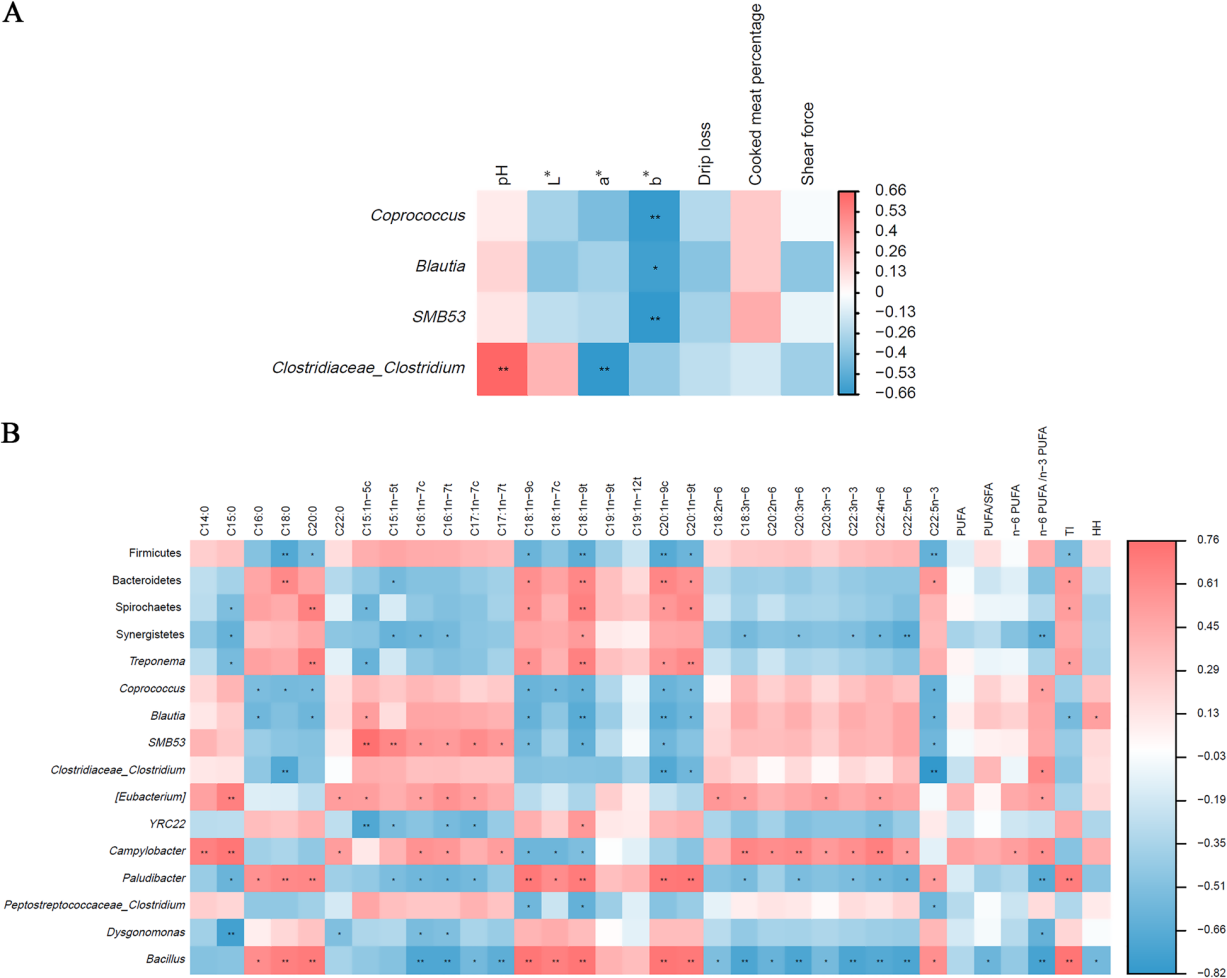
Spearman correlation between differentiated microbiota and meat quality indexes of spent hens. The red and blue colors represent a positive and a negative correlation, respectively (*n* = 8). ^*^P < 0.05, ^**^P < 0.01, |R| > 0.6. CON group, Conventional diet; CHF group, Conventional diet with the addition of 1% Chinese herbal formula; PUFA, Polyunsaturated fatty acids; SFA, Saturated fatty acids; TI, Thrombogenic index; HH, Hypocholesterolemic/hypercholesterolemic ratio

As shown in Fig. 7A, *Clostridiaceae_Clostridium* had a positive correlation (*P* < 0.05) with pH value. Additionally, negative correlations (*P* < 0.05) were observed between *Clostridiaceae_Clostridium* with a* value; *Coprococcus*, *Blautia*, and *SMB53* with b* value.

As displayed in Fig. 7B, positive correlations (*P* < 0.05) were included between Bacteroidetes with C18:0, C18:1n-9c, C18:1n-9t, C20:1n-9c, C20:1n-9t, C22:5n-3, and TI; Spirochaetes with C18:1n-9c, C18:1n-9t, C20:0, C20:1n-9c, C20:1n-9t, and TI; Synergistetes with C18:1n-9t; *Treponema* with C18:1n-9c, C18:1n-9t, C20:0, C20:1n-9c, C20:1n-9t, and TI; *Coprococcus* with n-6/n-3 PUFA; *Blautia* with C15:1n-5c and HH; *SMB53* with C15:1n-5c, C15:1n-5t, C16:1n-7c, C16:1n-7t, C17:1n-7c, and C17:1n-7t; *Clostridiaceae_Clostridium* with n-6/n-3 PUFA; *[Eubacterium]* with C15:0, C15:1n-5c, C16:1n-7c, C16:1n-7t, C17:1n-7c, C18:2n-6, C18:3n-6, C20:3n-3, C22:0, C22:4n-6, and n-6/n-3 PUFA; *YRC22* with C18:1n-9t; *Campylobacter* with C14:0, C15:0, C16:1n-7c, C16:1n-7t, C17:1n-7t, C18:3n-6, C20:2n-6, C20:3n-6, C20:3n-3, C22:0, C22:3n-3, C22:4n-6, C22:5n-6, n-6 PUFA, and n-6/n-3 PUFA; *Paludibacter* with C16:0, C18:0, C18:1n-9c, C18:1n-7c, C18:1n-9t, C20:0, C20:1n-9c, C20:1n-9t, C22:5n-3, and TI; *Bacillus* with C16:0, C18:0, C18:1n-9c, C18:1n-7c, C18:1n-9t, C20:0, C20:1n-9c, C20:1n-9t, C22:5n-3 and TI.

Furthermore, negative correlations (*P* < 0.05) were included between Firmicutes with C18:0, C18:1n-9c, C18:1n-9t, C20:0, C20:1n-9c, C20:1n-9t, C22:5n-3, and TI; Bacteroidetes with C15:1n-5t; Spirochaetes with C15:0 and C15:1n-5c; Synergistetes with C15:0, C15:1n-5t, C16:1n-7c, C16:1n-7t, C18:3n-6, C20:3n-6, C22:3n-3, C22:4n-6, C22:5n-6, and n-6/n-3 PUFA; *Treponema* with C15:0 and C15:1n-5c; *Blautia* with C16:0, C18:1n-9c, C18:1n-9t, C20:0, C20:1n-9c, C20:1n-9t, C22:5n-3 and TI; *SMB53* with C18:1n-9c, C18:1n-9t, C20:1n-9c, and C22:5n-3; *Clostridiaceae_Clostridium* with C18:0, C20:1n-9c, C20:1n-9t, and C22:5n-3; *YRC22* with C15:1n-5c, C15:1n-5t, C16:1n-7t, C17:1n-7c, and C22:4n-6; *Campylobacter* with C18:1n-9c, C18:1n-7c, and C18:1n-9t; *Paludibacter* with C15:0, C15:1n-5t, C16:1n-7c, C16:1n-7t, C17:1n-7c, C18:3n-6, C20:3n-6, C22:3n-3, C22:4n-6, C22:5n-6, and n-6/n-3 PUFA; *Peptostreptococcaceae_Clostridium* with C18:1n-9c, C18:1n-9t, and C22:5n-3; *Dysgonomonas* with C15:0, C16:1n-7c, C16:1n-7t, C22:0, and n-6/n-3 PUFA; *Bacillus* with C16:1n-7c, C16:1n-7t, C17:1n-7c, C17:1n-7t, C18:2n-6, C18:3n-6, C20:2n-6, C20:3n-6, C20:3n-3, C22:3n-3, C22:4n-6, C22:5n-6, PUFA/SFA, n-6/n-3 PUFA, and HH.

## Discussion

Spent hens are one of the most fundamental sources of poultry meat. Spent hen meat has poor quality, lower acceptability, and lower selling price compared with broiler meat [17]. Research has shown that feed quality is closely associated with muscle growth, protein synthesis, and fat deposition. In addition, modification of feed nutritional composition can significantly improve meat quality in chickens [18]. The current research revealed that adding CHF to the spent hens’ diet enhanced the quality, nutritional profile, and antioxidant capacity of the breast muscle, as well as reshaping the cecal microbiota.

Improving the meat quality of spent hens can enhance their economic value. In this study, dietary CHF supplementation reduced the shear force of spent hens’ breast muscles and then improved the tenderness, which may be related to the degradation of muscle fibers by several bioactive components in CHF, such as quercetin [19]. Regarding the meat color, deeper redness, lower brightness, and lower yellowness values indicate better-quality meat [20]. Our findings demonstrated that dietary CHF supplementation decreased the yellowness value (b*), suggesting that CHF might reduce the oxidation of myoglobin, resulting in a decrease in the accumulation of brown metmyoglobin [21]. Similarly, dietary fermented TM has been reported to decrease the b* value of the breast muscle of broilers [22]. Drip loss, cooked meat percentage, and DM reflect the water-holding capacity (WHC) of muscles. Previous studies found that dietary Chinese herbal additives, such as LJ [23] and fermented TM [22] (two herbs of the CHF), decreased drip loss of the meat in broilers. In our study, dietary CHF supplementation decreased drip loss while increasing cooked meat percentage and DM content, suggesting an enhancement in WHC and reduction in nutrient loss in the breast muscle of spent hens. These findings indicate that CHF addition in laying hens’ diet enhanced the meat quality of breast muscle of spent hens.

The fatty acid composition is associated with nutritional value and lipid health indices of meat [24]. An appropriate proportion of fatty acids, especially higher PUFA, is crucial to preventing cardiovascular, atherosclerosis, and other relevant chronic diseases [25]. Our study revealed that CHF supplementation elevated the PUFA/SFA ratio, which may be due to the fact that CHF supplementation increased the levels of C18:2n-6 and C18:3n-6. The C18:2n-6 is the substrate for generating n-6 PUFAs with more carbon atoms in the body [26]. Additionally, although the laying hens in the CHF group had a higher n-6/n-3 PUFA, they still met the healthy conditions (2.60, less than 4.0) [27]. HH and TI are standard lipid health indices, which are positively and negatively correlated with health, respectively [9]. In the current study, dietary CHF inclusion enhanced the HH index while decreasing the TI index, suggesting that dietary CHF enhanced the health-promoting value of breast muscle. It can be postulated that CHF administration enriched the metabolic pathways related to linoleic acid (C18:2n-6) metabolism and biosynthesis of unsaturated fatty acids.

Lipid metabolic disorders (including FLHS) and OS are the two main challenges faced by aging laying hens [7]. The occurrence and development of FLHS and OS are caused by various pathological triggers. The synergistic effects of multiple components and targets of CHF can largely address the complex pathological mechanisms in the body. The network pharmacology analysis revealed that the compounds in CHF, including luteolin (A1), kaempferol (A2), quercetin (A3), dan-shexinkum d (SM32), arachidonic acid (LJ5), isorhamnetin (LJ6), choline (TM3), and others were related to the targets associated with FLHS and OS, such as PPARG, PPARD, PPARA, APOB, NFE2L2, SOD1, HMOX1, NQO1, etc. It was found that compounds such as quercetin and kaempferol can enhance lipid metabolism and reduce the oxidative stress [28]. FLHS may lead to an energy metabolism imbalance between the liver and muscle [29], manifested as muscle atrophy. It has been reported that magnesium lithospermate B from SM (one herb of CHF) could inhibit the skeletal muscle degradation caused by a high-fat diet in a mouse model [30]. Our results suggested that dietary CHF supplementation tended to increase PUFA, which may be related to CHF enhancing the gene expression of *SREBP1*, *PPARγ*, and *CPT1A* in breast muscle. SREBP1, as an auxiliary regulatory factor of fatty acid synthase, can promote the generation of fatty acids [31]. The expression of *PPARγ* in muscles decreases with age, leading to carbohydrate-lipid metabolism disorders [32]. Dietary CHF could improve fatty acid metabolism by upregulating several genes related to lipid metabolism, such as *PPARγ* and *CPT1* in breast muscle. Spent hens’ breast muscles exhibit a higher content of lipid peroxides, potentially leading to a decrease in the sensory attributes and nutritional value of the meat [2]. This occurrence is closely associated with the oxidative damage observed in muscles during aging [33]. Previous studies showed that dietary LJ [23], LL [34], and fermented TM [22] (herbs from CHF) separately can improve the chicken muscles’ antioxidant function. In the present study, the addition of CHF significantly enhanced the antioxidant function of breast muscle in spent hens by increasing the GPX activity and upregulating *Nrf2*, *HO-1*, *CAT*, *SOD2*, and *GPX1* expression. The above findings demonstrate that dietary CHF could improve the meat quality of spent hens by ameliorating lipid metabolism and OS dysfunction.

The gut microbiota is closely associated with the metabolism of host macronutrients through the gut-muscle axis [35], and optimizing the gut microbiota can improve meat quality [36]. The composition and structure of the gut microbiota in aged laying hens undergo significant changes [37]. Firmicutes and Bacteroidetes account for > 90% of intestinal microbiota, and the F/B ratio has a significant impact on maintaining normal intestinal homeostasis [38]. In this study, dietary CHF supplementation increased the abundance of Firmicutes and decreased the abundance of Bacteroidetes in the cecum of spent hens. This finding suggests that CHF may partially increase F/B, improve intestinal digestion and absorption functions, and enhance the health status of laying hens. Previous studies also reported that adding TM [39] and SM polysaccharides [40] to the broilers’ diet increases the Firmicutes and decreases the Bacteroidetes abundances in the ileum and cecum. It is noteworthy that an increase in F/B not only enhances the intestinal health of hens but also positively affects muscle quality. In this study, the addition of CHF enhanced abundances of *Coprococcus*, *Clostridiaceae_Clostridium*, *Blautia*, and *SMB53* belong to the Firmicutes phylum, which were negatively associated with the b* value and positively associated with the n-6/n-3 PUFA of breast muscle. Furthermore, *Blautia* was negatively correlated with TI and positively correlated with HH. Compared with the control group, CHF supplementation reduced the abundance of Bacteroidetes (including *Paludibacter*), which was negatively correlated with n-6/n-3 PUFA and positively correlated with TI. Similarly, *Coprococcus* [41], *Clostridium* [41], *Blautia* [35], and *SMB53* [42] may affect the PUFA profiles in breast muscle. This may be attributed to the fact that, compared to Bacteroidetes, Firmicutes exhibit superior fermentation and metabolic capabilities for carbohydrates and lipids [38]. For instance, *Lactobacillus* *johnsonii* belonging to the Firmicutes phylum have been shown to upregulate the gene expression of liver *PPARγ* and *SREBP1* [43]. Overall, dietary CHF supplementation may enhance the muscle quality and nutritional value of breast muscle by optimizing the gut microbiota structure of spent hens.

## Conclusion

In summary, dietary 1.0% CHF (0.30% LJ + 0.20% SM + 0.25% LL + 0.25% TM) supplementation could improve the breast muscle quality and nutritional value of spent hens. Moreover, CHF supplementation enhanced the antioxidant function and lipid metabolism in breast muscle and improved the cecal microbiota structure of spent hens. This study will provide a scientific basis for further exploring CHF as a dietary supplement to ensure better health and enhance the economic value of the meat from spent hens.

## Supplementary Information

Additional file 1: Table S1. Ingredients and nutrient composition of the conventional diet. Table S2. Specific primers for real-time PCR analysis. Table S3. The compounds of four Chinese herbs. Table S4. The targets of four Chinese herbs. Fig. S1. Fatty acid (A–C) and amino acid (D–F) distribution in breast muscle of spent hens. Fig. S2. Protein-protein interaction of potential targets. Fig. S3. The α- and β-diversity indices of cecal microbiota of spent hens. Fig. S4. The Firmicutes/Bacteroidetes ratio of cecal microbiota of spent hens.

## Data Availability

The data used to support the results of the current study are available from the corresponding authors upon request.

## Abbreviations

AI: Atherogenic index
CHF: Chinese herbal formula
CP: Crude protein
DFA: Desirable fatty acids
DM: Dry matter
FLHS: Fatty liver hemorrhagic syndrome
HH: Hypocholesterolemia/hypercholesterolemia
HPI: Health promotion index
IMF: Intramuscular fat
LJ: *Leonurus japonicus* Houtt.
LL: *Ligustrum lucidum* Ait.
MUFA: Monounsaturated fatty acids
OS: Oxidative stress
PPI: Protein-protein interaction
PUFA: Polymonounsaturated fatty acids
SFA: Saturated fatty acids
SM: *Salvia miltiorrhiza* Bge.
TI: Thrombopoiesis index
TM: *Taraxacum mongolicum* Hand.-Mazz.
