# Supplementary material for: Metabolomics, network pharmacology, and microbiome analyses uncover the mechanisms of the Chinese herbal formula for the improvement of meat quality in spent hens
Source: J Anim Sci Biotechnol. 2025 Feb 3;16:17. doi: 10.1186/s40104-025-01150-8 (PMC11789354; doi:10.1186/s40104-025-01150-8)

**Metabolomics, network pharmacology, and microbiome analyses uncover the mechanisms of the Chinese herbal formula for the improvement of meat quality in spent hens**

Zhihua Li ^1,2^, Md. Abul Kalam Azad ^2^, Chengwen Meng ^2^, Xiangfeng Kong ^2,3^*, Jue Gui ^2^, Wenchao Lin ^2^, Yadong Cui ^3^, Wei Lan ^3^, Qinghua He ^1*^

^1^ Department of Food Science and Engineering, College of Chemistry and Environmental Engineering, Shenzhen University, Shenzhen, China

^2^ Hunan Provincial Key Laboratory of Animal Nutritional Physiology and Metabolic Process, National Engineering Laboratory for Pollution Control and Waste Utilization in Livestock and Poultry Production, Institute of Subtropical Agriculture, Chinese Academy of Sciences, Changsha, China

^3^ School of Biology and Food Engineering, Fuyang Normal University, Fuyang, China

* Corresponding authors.

E-mail address: qinghua.he@szu.edu.cn (Q. He), nnkxf@isa.ac.cn (X. Kong).

**Table S1** Ingredients and nutrient components of the conventional diet, % air-dried

| Ingredients | Content | Nutrient component^2^ | Content |
| --- | --- | --- | --- |
| Corn | 62.59 | Metabolizable energy (MJ/kg) | 11.25 |
| Soybean meal | 23.88 | Crude protein | 14.23 |
| Limestone powder | 7.94 | Ether extract | 9.66 |
| Soybean oil | 0.49 | Calcium | 3.51 |
| Methionine | 0.10 | Phosphorus | 0.34 |
| Premix^1^ | 5.0 | Lysine | 0.71 |
| Total | 100.00 | Methionine | 0.37 |

^1^The premix provided the following per kg of diets: vitamin A 7,000 IU, vitamin D_3_ 2,500 IU, vitamin E 24 mg, vitamin Κ_3_ 9 mg, VB_1_ 3.15 mg, VB_2_ 10 mg, VB_6_ 7 mg, VB_12_ 0.35 mg, nicotinic acid 50 mg, D-pantothenic acid 25 mg, folic acid 2.5 mg, D-biotin 0.25 mg, choline chloride 450 mg, Fe (as ferrous sulfate) 100 mg, Cu (as copper sulfate) 15 mg, Mn (as manganese sulfate) 90 mg, Zn (as zinc sulfate) 100 mg, I (as potassium iodide) 3.5 mg, Se (as sodium selenite) 0.45 mg, and phytase 150 IU.

^2^Crude protein and ether extract were measured values, while the others were calculated values.

**Table S2** Specific primers for real-time PCR analysis

| Gene name | GenBank accession no. | Sequence (5′→3′) | Product size (bp) |
| --- | --- | --- | --- |
| *β-actin* | NM_205518.1 | F: ATGAAGCCCAGAGCAAAAGA  R: GGGGTGTTGAAGGTCTCAAA | 223 |
| *ACC* | NM_205505.1 | F: GTTGTGGTTGGCAGAGCAAG  R: GCACCAAACTTGAGCACCTG | 284 |
| *CAT* | NM_001031215.2 | F: AGATGGCGTATGACCCTAGC  R: CCTCTGATAATTGGCCACGC | 173 |
| *CD36* | NM_001030731.1 | F: ACCAGACCAGTAAGACCGTG  R: GTCTAGGACTCCAGCCAGTG | 152 |
| *CPT1A* | NM_001012898.1 | F: GGGTTGCCCTTATCGTCACA  R: TACAACATGGGCTTCCGTCC | 151 |
| *FAS* | NM_205155.3 | F: TGAAGGACCTTATCGCATTGC  R: GCATGGGAAGCATTTTGTTGT | 96 |
| *FFAR3b* | NM_001318430.1 | F: GCACTCTCTTTATGGCTGCC  R: TCACCCCATAACCACCTTCC | 219 |
| *FFAR4* | XM_003641481.6 | F: GTGCTGCCAACTACCTTGTC  R: GGCCGAGAGAGAGACAATGA | 179 |
| *GPX1* | NM_001277853.2 | F: ATGTTCGAGAAGTGCGAGGT  R: AGTTCCAGGAGACGTCGTTG | 160 |
| *GR42L5* | NM_001318424.1 | F: GCACTCTCTTTATGGCTGCC  R: GGATTCCCTGGTCTTGGTCA | 242 |
| *HO-1* | NM_205344.1 | F: ATGCCTACACCCGCTATTTG  R: ATCTCAAGGGCATTCATTCG | 178 |
| *Keap1* | MN416132.1 | F: CATCAACTGGGTGCAGTACG  R: AGGGTGAGGTCCTGGAAGAT | 183 |
| *NQO1* | NM_001277619.1 | F: AAGAAGATTGAAGCGGCTGA  R: GCATGGCTTTCTTCTTCTGG | 166 |
| *Nrf2* | NM_205117.1 | F: CCACCCTAAAGCTCCATTCA  R: ATTCTTGCCTCTCCTGCGTA | 217 |
| *PPARα* | NM_001001464.1 | F: CAAACCAACCATCCTGACGAT  R: GGAGGTCAGCCATTTTTTGGA | 64 |
| *PPARγ* | NM_001001460.2 | F: AGGGCGATCTTGACAGGAAA  R: TAATCTCCTGCACTGCCTCC | 190 |
| *SOD1* | NM_205064.1 | F: ATTACCGGCTTGTCTGATGG  R: CCTCCCTTTGCAGTCACATT | 173 |
| *SOD2* | NM_204211.1 | F: CCTTCGCAAACTTCAAGGAG  R: CCAGCAATGGAATGAGACCT | 162 |
| *SREBP1* | AY029224.1 | F: GTCGGCGATCCTGAGGAA  R: CTCTTCTGCACGGCCATCTT | 105 |

*ACC*, acetyl-CoA carboxylase; *CAT*, catalase; *CD36*, CD36 molecule; *CPT1A*, carnitine palmitoyl transferase 1A; *FAS*, fatty acid synthase; *FFAR3b*, free fatty acid receptor 3b; *FFAR4*, free fatty acid receptor 4; *GPX1*, glutathione peroxidase 1; *GR42L5*: G-protein coupled receptor 42-like 5; *HO-1*, heme oxygenase 1; *Keap1*, Kelch-like ECH-associated protein 1; *NQO1*, NAD(P)H quinone dehydrogenase 1; *Nrf2*, NF-E2-related factor 2; *PPARα*, peroxisome proliferator activated receptor alpha; *PPARγ*, peroxisome proliferator activated receptor gamma; *SOD1*, superoxide dismutase 1; *SOD2*, superoxide dismutase 2; *SREBP1*, sterol regulatory element binding protein 1.

**Table S3** The compounds of four Chinese herbs

| Herb | Total numbers of compounds | Ingredients |
| --- | --- | --- |
| *Salvia miltiorrhiza* Bge. (SM) | 65 | 1,2,5,6-tetrahydrotanshinone (SM1), poriferasterol (SM2), poriferast-5-en-3beta-ol (SM3), isoimperatorin (SM4), sugiol (SM5), dehydrotanshinone II A (SM6), baicalin (SM7), digallate (SM8), α-amyrin (SM9), 5,6-dihydroxy-7-isopropyl-1,1-dimethyl-2,3-dihydrophenanthren-4-one (SM10), 2-isopropyl-8-methylphenanthrene-3,4-dione (SM11), 3α-hydroxytanshinone IIa (SM12), (E)-3-[2-(3,4-dihydroxyphenyl)-7-hydroxy-benzofuran-4-yl]acrylic acid (SM13), 4-methylenemiltirone (SM14), 2-(4-hydroxy-3-methoxyphenyl)-5-(3-hydroxypropyl)-7-methoxy-3-benzofurancarboxaldehyde (SM15), 6-o-syringyl-8-o-acetyl shanzhiside methyl ester (SM16), formyltanshinone (SM17), 3-beta-hydroxymethyllenetanshiquinone (SM18), methylenetanshinquinone (SM19), przewalskin a (SM20), przewalskin b (SM21), przewaquinone B (SM22), przewaquinone c (SM23), (6S,7R)-6,7-dihydroxy-1,6-dimethyl-8,9-dihydro-7H-naphtho[8,7-g]benzofuran-10,11-dione (SM24), przewaquinone f (SM25), sclareol (SM26), tanshinaldehyde (SM27), danshenol B (SM28), danshenol A (SM29), salvilenone (SM30), cryptotanshinone (SM31), dan-shexinkum d (SM32), danshenspiroketallactone (SM33), deoxyneocryptotanshinone (SM34), dihydrotanshinlactone (SM35), dihydrotanshinone I (SM36), epidanshenspiroketallactone (SM37), C09092 (SM38), isocryptotanshi-none (SM39), isotanshinone II (SM40), manool (SM41), microstegiol (SM42), miltionone I (SM43), miltionone II (SM44), miltipolone (SM45), miltirone (SM46), miltirone II (SM47), neocryptotanshinone II (SM48), neocryptotanshinone (SM49), 1-methyl-8,9-dihydro-7H-naphtho[5,6-g]benzofuran-6,10,11-trione (SM50), prolithospermic acid (SM51), (2R)-3-(3,4-dihydroxyphenyl)-2-[(Z)-3-(3,4-dihydroxyphenyl)acryloyl]oxy-propionic acid (SM52), (Z)-3-[2-[(E)-2-(3,4-dihydroxyphenyl)vinyl]-3,4-dihydroxy-phenyl]acrylic acid (SM53), salvianolic acid g (SM54), salvianolic acid j (SM55), salvilenone I (SM56), salviolone (SM57), NSC 122421 (SM58), (6S)-6-hydroxy-1-methyl-6-methylol-8,9-dihydro-7H-naphtho[8,7-g]benzofuran-10,11-quinone (SM59), tanshindiol B (SM60), przewaquinone E (SM61), tanshinone IIA (SM62), (6S)-6-(hydroxymethyl)-1,6-dimethyl-8,9-dihydro-7H-naphtho[8,7-g]benzofuran-10,11-dione (SM63), tanshinone VI (SM64), luteolin (A1) |
| *Leonurus japonicus* Houtt. (LJ) | 8 | Galeopsin (LJ1), ZINC04073977 (LJ2), preleoheterin (LJ3), iso-preleoheterin (LJ4), arachidonic acid (LJ5), isorhamnetin (LJ6), kaempferol (A2), quercetin (A3) |
| *Ligustrum lucidum* Ait. (LL) | 13 | beta-sitosterol (LL1), taxifolin (LL2), lucidumoside D (LL3), lucidumoside D_qt (LL4), (20S)-24-ene-3β,20-diol-3-acetate (LL5), eriodictyol (LL6), syringaresinol diglucoside_qt (LL7), lucidusculine (LL8), olitoriside (LL9), olitoriside_qt (LL10), luteolin (A1), kaempferol (A2), quercetin (A3) |
| *Taraxacum mongolicum* Hand.-Mazz (TM) | 8 | Taraxasterol (TM1), chrysanthemaxanthin (TM2), choline (TM3), esculetin (TM4), scopoletin (TM5), caffeicacid (TM6), taraxerol (TM7), flavoxanthin (TM8) |

**Table S4** The targets of four Chinese herbs

| Herbs | Total numbers of targets | Elements |
| --- | --- | --- |
| LJ LL SM TM | 8 | PTGS2, RXRA, ACHE, PGR, PTGS1, AR, PPARG, ESR1 |
| LJ LL SM | 49 | F2, CHRM1, SLC6A2, CHRM2, ADRB2, SLC6A4, GABRA1, CHRM3, PDE3A, ADRA1B, F10, HSP90AA1, PRKACA, CALM2, NCOA2, DPP4, AKR1B1, PRSS1, TOP2B, KCNH2, SCN5A, F7, NOS3, RELA, EGFR, AKT1, VEGFA, CCND1, BCL2, BCL2L1, FOS, CDKN1A, CASP9, MMP2, MMP9, MAPK1, IL10, RB1, JUN, IL6, AHSA1, CASP3, TP53, NFKBIA, CDK4, NOS2, GSK3B, CDK2, CCNA2 |
| LJ LL TM | 13 | BAX, XDH, PRKCA, HMOX1, CYP3A4, PRKCB, NR1I2, IFNG, ALOX5, NQO1, PPARA, CRP, AKR1C3 |
| LJ SM | 8 | ADRA2A, ADRA2B, SLC6A3, PTPN1, ESR2, MAPK14, CHEK1, NCOA1 |
| LJ LL | 99 | MAOB, MMP3, EIF6, PLAU, EGF, CD40LG, ELK1, POR, ODC1, CASP8, TOP1, RAF, SOD1, MMP1, HIF1A, STAT1, RUNX1T1, CDK1, HSPA5, ERBB2, ACACA, CYP1A2, CAV1, MYC, F3, GJA1, CYP1A1, ICAM1, IL1B, CCL2, SELE, VCAM1, PTGER3, CXCL8, BIRC5, DUOX2, HSPB1, TGFB1, SULT1E1, MGAM, IL2, CYP1B1, CCNB1, PLAT, THBD, SERPINE1, COL1A1, PTEN, IL1A, MPO, TOP2A, NCF1, ABCG2, HAS2, GSTP1, NFE2L2, PARP1, AHR, PSMD3, SLC2A4, COL3A1, CXCL11, CXCL2, DCAF5, NR1I3, CHEK2, INSR, CLDN4, PPARD, HSF1, CXCL10, CHUK, SPP1, RUNX2, RASSF1, E2F1, E2F2, ACPP, CTSD, IGFBP3, IGF2, IRF1, ERBB3, PON1, DIO1, PCOLCE, NPEPPS, HK2, NKX3-1, RASA1, PRXC1A, GSTM1, GSTM2, PTGES, GABRA2, IKBKB, MAPK8, PPP3CA, SLPI |
| LL SM | 11 | PIK3CG, DRD1, CHRM4, HTR2A, GABRA5, ADRA1D, CHRNA2, OPRM1, CA2, NR3C2, APP |
| SM TM | 1 | NR3C1 |
| LJ | 30 | ADRB1, ADH1C, GRIA2, PIK3CG, CDKN2A, OLR1, GYRB, TRPV1, RXRG, G6PD, TNFRSF1A, PECAM1, PLA2G4A, SELP, GLB1, ALDH2, ABCA1, ALDH3A1, UCP2, C1R, CETP, ABCG1, ABCC4, KCNK10, TNFRSF1B, PTGES2, KCNK2, COL1A2, PIM1, PYGM |
| SM | 19 | CHRM5, HTR3A, OPRD1, CHRNA7, IGHG1, DRD2, TNF, ADRA2C, PIM1, KCNMA1, STAT3, EDN1, LACTBL1, DRD5, HTR1A, GABRA2, GABRA3, HTR2C, FASN |
| LL | 19 | GABRA3, CHRNA7, MAP2, MAC1, DGAT2, MTTP, APOB, MDM2, PCNA, CASP7, MCL1, TYR, IL4, XIAP, NUF2, ADCY2, MET, CDK2A, GYRB |
| TM | 101 | VDR, CYP27B1, GC, SNW1, TRPV3, OPRK1, TRPM8, TRPA1, NFKB1, CYP24A1, GPBAR1, SNAI2, MED1, SNAI1, CALB1, FGF23, GFI1, LANCL2, WNT4, TCF3, KL, CYP2R1, PML, B4GALT1, S100G, CYP27A1, KANK2, IRX5, NR1H4, TRIM24, ADA, ITFG2, DGUOK, ADORA1, PLD1, PCYT1A, PCYT1B, PHOSPHO1, PLD2, BCHE, COLQ, SLC5A7, NRG1, DMGDH, SIX3, RAP1GAP, NAPEPLD, FSCN1, PRSS12, KLF5, SLC44A4, ALDH7A1, DNM3, PLD4, ASCL1, GPLD1, ENPP6, CDH8, PLD3, PODXL, FNTA, AGRN, CHKA, CHDH, ATP8B1, GRIN1, DHFR, LRP2, PTGDR2, PLA2G2A, GLO1, PTGR2, CRYZ, VKORC1, COMT, SEC14L3, PPP2CA, PPP2CB, SEC14L2, DGKA, SEC14L4, TPMT, RDH11, SRD5A1, RBP3, RETSAT, RDH13, DHRS3, RDH5, F12, ALDH1A3, RDH12, DHRS4, RBP1, ALDH1A1, RLBP1, RDH14, RDH8, ANXA1, LRAT, ALDH1A2 |

LJ, *Leonurus japonicus* Houtt.; SM, *Salvia miltiorrhiza* Bge.; LL, *Ligustrum lucidum* Ait.; TM, *Taraxacum mongolicum* Hand.-Mazz.

**Fig. S1.** Fatty acid (A−C) and amino acid (D−F) distribution in breast muscle of spent hens. The PCA (A), permutation test of the OPLS-DA model (B), and volcano plots (C) of differential fatty acids for the CHF vs. CON groups (*n* = 8). The PCA (D), permutation test of the OPLS-DA model (E), and volcano plots (F) of differential amino acids for the CHF vs. CON groups. CON group, Conventional diet; CHF group, Conventional diet with the addition of 1% Chinese herbal formula; PCA, Principal component analysis; OPLS-DA, Orthogonal projections to latent structures-discriminant analysis.


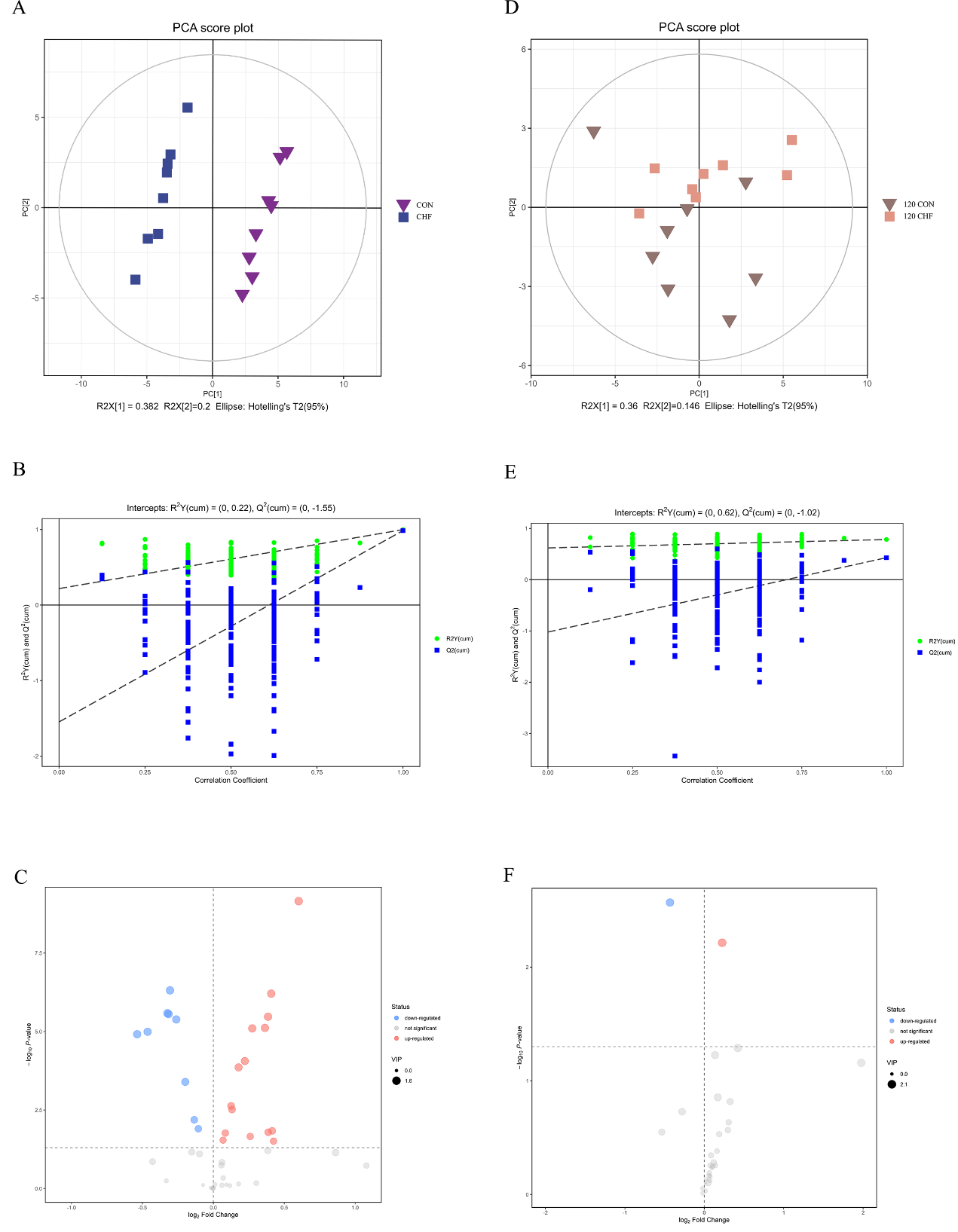


**Fig. S2.** Protein-protein interaction of potential targets.


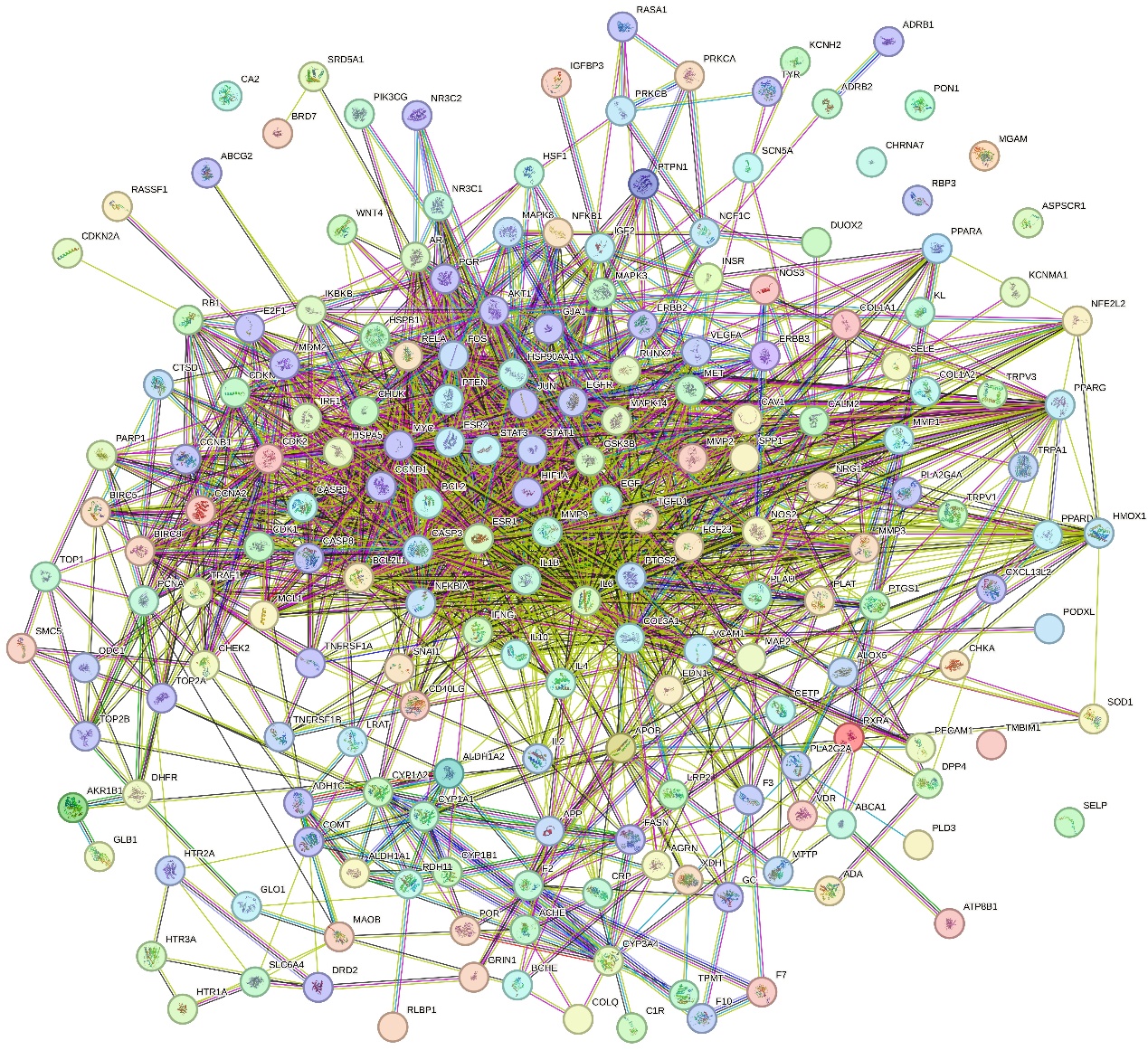


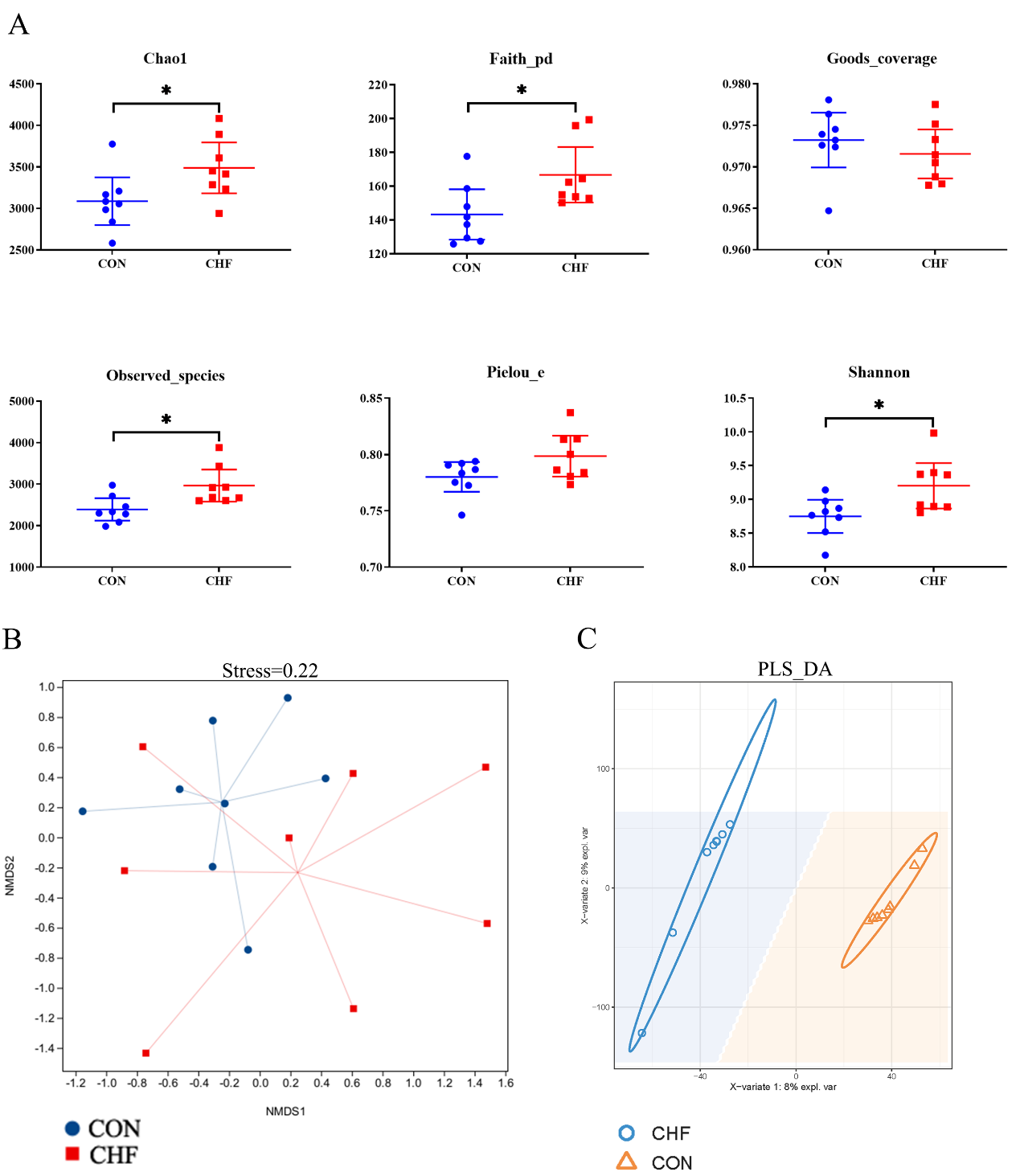
**Fig. S3.** The α- and β-diversity indices of cecal microbiota of spent hens. Alpha-diversity (A), non-metric multidimensional scaling (NMDS) analysis (B), and partial least squares discriminant analysis (PLS-DA) analysis (C) of cecal microbiota. CON group, Conventional diet; CHF group, Conventional diet with the addition of 1% Chinese herbal formula. The asterisk suggests significant differences (*n* = 8).

**Fig. S4.** The Firmicutes/Bacteroidetes ratio of cecal microbiota of spent hens. CON group, Conventional diet; CHF group, Conventional diet with the addition of 1% Chinese herbal formula; F/B, Firmicutes/Bacteroidetes.


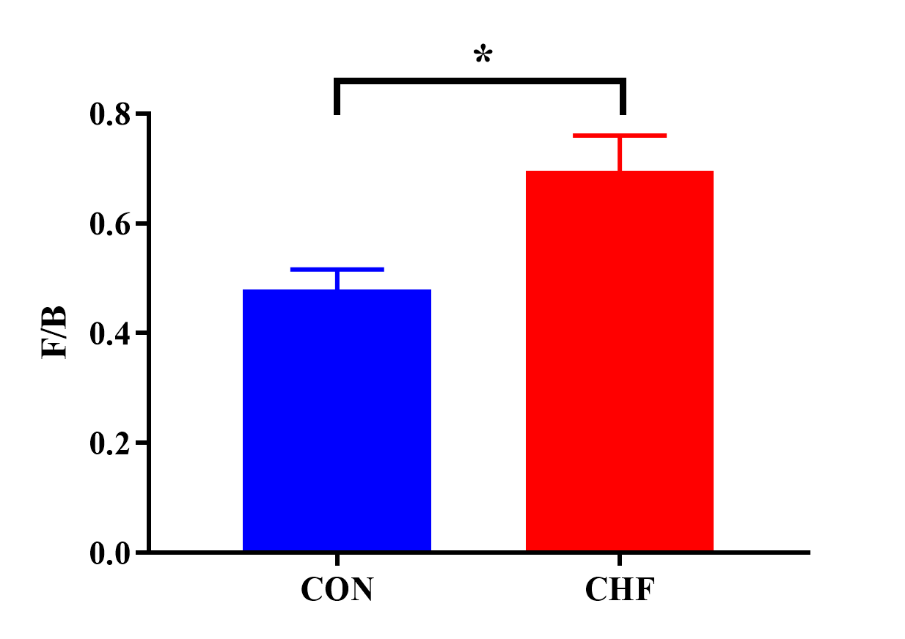

Supplement: Supplementary file 1 — Additional file 1: Table S1. Ingredients and nutrient composition of the conventional diet. Table S2. Specific primers for real-time PCR analysis. Table S3. The compounds of four Chinese herbs. Table S4. The targets of four Chinese herbs. Fig. S1. Fatty acid (A–C) and amino acid (D–F) distribution in breast muscle of spent hens. Fig. S2. Protein-protein interaction of potential targets. Fig. S3. The α- and β-diversity indices of cecal microbiota of spent hens. Fig. S4. The Firmicutes/Bacteroidetes ratio of cecal microbiota of spent hens. [file 40104_2025_1150_MOESM1_ESM.docx]
